# Descriptions of immature stages of four species of the genera *Graptus*, *Peritelus*, *Philopedon*, and *Tanymecus* and larval instar determination in *Tanymecus* (Coleoptera, Curculionidae, Entiminae)

**DOI:** 10.3897/zookeys.813.30336

**Published:** 2019-01-07

**Authors:** Rafał Gosik, Peter Sprick, Michael G. Morris

**Affiliations:** 1 Department of Zoology, Maria Curie–Skłodowska University, Akademicka 19, 20–033 Lublin, Poland Maria Curie-Skłodowska University Lublin Poland; 2 Curculio-Institute e.V. (CURCI), Weckenstraße 15, 30451 Hannover, Germany Curculio-Institute Hannover Germany; 3 Natural History Museum, Cromwell Road, London SW7 5BD, United Kingdom Natural History Museum London United Kingdom

**Keywords:** Bionomics, Central European region, chaetotaxy, Curculionoidea, Entiminae, immature stages, larval instar determination, morphology, taxonomy, weevils

## Abstract

The mature larva and pupa of *Graptustriguttatustriguttatus* and the mature larva of *Peritelussphaeroides* are described for the first time. The larvae of *Philopedonplagiatum* and *Tanymecuspalliatus* are re-described. Five larval instars were determined in *Tanymecus*, thereby correcting doubtful data in the literature. The relationship between larval growth, number of larval instars, head width of the mature larva, and the adult weevil is explained using the example of *Tanymecus*. The nearly constant ratio of subsequent larval instars in head width ratio, termed “growth factor” and derived from Dyar’s ratio, is used for the determination of larval instars. Larval collecting and breeding data are discussed in relation to their significance for the clarification of life-cycles.

## Introduction

In this continued contribution on larvae of the subfamily Entiminae Schönherr, 1823 we describe or redescribe the mature larvae of four further species (e.g., Sprick and Gosik 2014; Gosik et al. 2016, 2017) and the pupa of one species. They represent four different tribes: Byrsopagini Lacordaire, 1863 (= Alophini LeConte, 1874), Cneorhinini Lacordaire, 1863, Peritelini Lacordaire, 1863, and Tanymecini Lacordaire, 1863 (Alonso-Zarazaga et al. 2017). They also allow some insight into the morphological diversity of Central European Entiminae larvae.

In the present paper we describe for the first time the mature larva and pupa of *Graptustriguttatus* (Fabricius, 1775). For this species, Van Emden (1952) provided a description of the first instar larva, eggs and oviposition habit. Dudich (1921) provided two host plant records, *Beta vulgaris* L. and *Symphytumofficinale* L., and some data about oviposition and egg morphology, but no relevant information about larval or pupal stages was given. For *Philopedonplagiatum* (Schaller, 1783) there is a description of the mature larva by Van Emden (1952), but only the head capsule and the right pedal lobe were illustrated. A study of distribution and biology of this species in Great Britain was published by Morris (1987).

From the tribe Peritelini we describe the mature larva of *Peritelussphaeroides* Germar, 1824 for the first time. The pupa was already described by Gosik and Sprick (2013). We do not know of any description of a Central European species in this tribe. Van Emden (1952) and Rosenstiel (1987) characterized the larvae of two North American Peritelini genera, *Nemocestes* Van Dyke, 1936 and *Peritelinus* Casey, 1888.

Despite the frequency and abundance of *Tanymecuspalliatus* (Fabricius, 1787), and the good characterization of its development (Hoffmann 1963; Dieckmann 1983), there is no detailed description of the larval instars of this species. Only Znamenskij (1927), in his keys to soil insects, depicted the habitus and last abdominal segment. But this source is not readily available, and we received only a few pages of this work through the kindness of Vitaliy Nazarenko. These studies had been carried out after damage by this species to sunflower and beet fields in Ukraine and southern Russia in the 1920s and 1930s. To our knowledge, the most complete description of a *Tanymecus* Germar, 1817 larva was published by Catrinici (1944) for *T.dilaticollis*. It is supplemented by Van Emden (1952) with a description of the mature larva of *T.palliatus* (without figures) and a description of the North American *T.confusus* (“or very near”). The pupa of *T.palliatus* was already described by Gosik and Sprick (2013).

The aim of this paper is to describe the mature larvae of the four Entiminae species mentioned before and to give some examples about how to use data from larval descriptions for the determination of larval instars and for the study of life-cycles. An important prerequisite for studying life-cycles is to have correctly identified larvae, which is often difficult and a main reason why life-cycles of Entiminae weevils, apart from some noxious *Otiorhynchus* and *Sitona* species, are usually little known.

## Materials

Specimens of three of the four species studied were collected in the field under certain plants and usually at the same sites where adults were previously collected. Larval instars of the fourth species, *Peritelussphaeroides* Germar, 1824, were obtained in captivity by breeding in an air-conditioned room (see Gosik et al. 2016). Two searches for preimaginal stages at the field site where adults of this species were known to occur, were unsuccessful. Number of specimens examined, date and places of collecting are given ahead of the description of each species. As “mature” we regard the larvae with the largest head capsule widths (most closely corresponding to head size of pupa and adult of the species). We also take into consideration results of measurements (if available) provided by other authors.

## Methods

All specimens studied were fixed in 75% ethanol and examined under an optical stereomicroscope (Olympus SZ 60 and SZ11) with calibrated oculars. Measurements of larval instars were made for: body length (BL), body width (BW) (usually at abdominal segment I or II), width (HW) and height (HH) of the head capsule (see Fig. 18). In pupae, body length (BL), body width (BW) (at the level of middle legs) and width of pronotum (= thorax) (THW) were measured.

The observations and measurements were conducted using a light compound microscope with calibrated oculars. Drawings and outlines were made using a drawing tube (MNR–1) installed on a stereomicroscope (Ampliwal) and processed by computer software (Corel Photo-Paint X7, Corel Draw X7). Photos were taken with an Olympus E-M10 or using an Olympus BX63 microscope and processed by Olympus cellSens Dimension software. The larvae selected for pictures using SEM (scanning electron microscope) were at first dried in absolute ethyl alcohol (99.8%), rinsed in acetone, treated by CPD procedure (critical point drying) and then gold-plated. For the examination of selected structures a TESCAN Vega 3 SEM was used. General terminology and chaetotaxy follow Anderson (1947), May (1994), Marvaldi (1997, 1998a, 1998b, 1999) and Skuhrovec et al. (2015), with terminology for antennae following Zaharuk (1985), May (1994) and Marvaldi (1998).

**Morphological abbreviations**:

**Abd. 1–10** – abdominal segments 1–10, **Th. 1**–**3** – thoracic segments 1–3, **at** – antenna, **clss** – clypeal sensorium, **st** – stemmata, **Se** – sensorium, **sa** – sensillum ampullaceum, **sb** – sensillum basiconicum, **lr** – labral rods, **ur** – urogomphus; setae: ***als*** – anterolateral, ***ams*** – anteromedial, ***as*** – alar (larva), ***as*** – apical (pupa), ***cls*** – clypeal, ***d*** – dorsal, ***des*** – dorsal epicranial, ***dms*** – dorsal malar, ***ds*** – discal (pupa), ***ds*** – dorsal (larva), ***eps*** – epipleural, ***es*** – epistomal, ***eus*** – eusternal, ***fes*** – femoral, ***fs*** – frontal, ***les*** – lateral epicranial, ***ligs*** – ligular, ***lrs*** – labral, ***ls*** – lateral, ***lsts*** – laterosternal, ***mbs*** – malar basiventral, ***mds*** – mandibular, ***mes*** – median, ***mps*** – maxillary palp, ***os*** – orbital, ***pas*** – postantennal, ***pda*** – pedal, ***pds*** – postdorsal, ***pls*** – posterolateral, ***pes*** – postepicranial, ***pfs*** – palpiferal, ***pms*** – postlabial, ***prms*** – prelabial, ***prns*** – pronotal, ***prs*** – prodorsal, ***ps*** – pleural, ***rs*** – rostral, ***sos*** – superorbital, ***ss*** – spiracular, ***stps*** – stipal, ***sts*** – sternal, ***ts*** – terminal, ***v*** – ventral (pupa), ***ves*** – ventral (larva), ***vms*** – ventral malar, ***vs*** – vertical.

We follow Trnka et al. (2015) and Skuhrovec et al. (2015) who counted in weevils 3 pairs of *ams* and 2 pairs of *mes.* Position of the distal pair of *mes* is still questionable and some other authors (e.g. May 1994; Marvaldi 1998) reported for weevil larvae 2 pairs of *ams* and 3 pairs of *mes*, and they regarded *ams_1_*as*mes_3_*.

All these specimens are deposited in the collection of the Department of Zoology, Maria Curie-Skłodowska University (Lublin, Poland). In Table 4 the chaetotaxy of the larvae is given. If necessary, head width of adults was measured directly behind eyes.

Larval instar determination is based on Dyar’s law (Dyar 1890), which had been developed and refined by Leibee et al. (1980), Rowe and Kok (1985) and Sprick and Gosik (2014). For the instar determination only data of L_1_ larvae and of mature larvae are needed. We explain in several steps how this method works, define the ‘growth factor’ (based on Dyar’s ratio), use it in all detail in *Tanymecusdilaticollis* and show how to find the best approximation of the factor that determines larval growth.

## Results

### Graptus triguttatus triguttatus

Taxon classificationAnimaliaColeopteraCurculionidae

#### Specimens examined.

**5 premature larvae**: Germany, Niedersachsen, Braunschweig, apple orchard and meadow in the area of Julius-Kühn-Institut (JKI), all under *Plantagolanceolata* L.; 10.01.2013: 3 ex., 18.04.2013: 1 ex., 24.05.2013: 1 ex.

**22 mature and penultimate instar larvae**: same site as previously noted, 10.1.2013: 5 ex., 18.04.2013: 7 ex., 03.05.2013: 7 ex., 24.05.2013: 1 ex., 10.06.2013: 2 ex.

**3 pupae**: Germany, Niedersachsen, Braunschweig, JKI, apple orchard, meadow; 2 mature larvae, collected on 24.05. and 10.06.2013, both had developed into the pupal stage in breeding boxes in the laboratory in Hannover on 20.06.2013. Another mature larva from the same site, collected on 03.05.2013, developed into the pupal stage before 27.05.2013.

#### Description of the mature larva.

Body length: 6.8–8.5 mm, body width: 3.0–3.4 mm, head width: 1.53–1.55 mm, head height: 1.35–1.37 mm.

**Figures 1–12.**
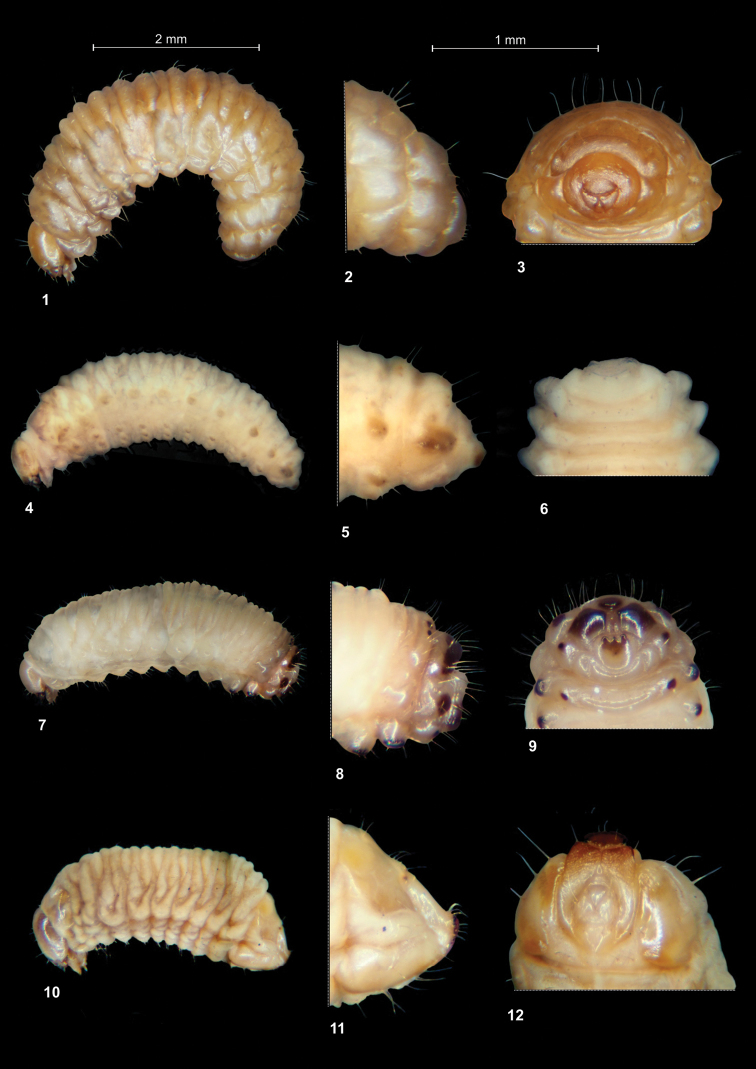
*Graptustriguttatus* mature larva. **1** Habitus **2** Last abdominal segments lateral view **3** Last abdominal segments ventral view *Peritelussphaeroides* mature larva. **4** Habitus **5** Last abdominal segments lateral view **6** Last abdominal segments ventral view *Philopedonplagiatum* mature larva **7** Habitus **8** Last abdominal segments lateral view **9** Last abdominal segments ventral view *Tanymecuspalliatus* mature larva **10** Habitus **11** Last abdominal segments lateral view **12** Last abdominal segments ventral view.

**Figures 13–17.**
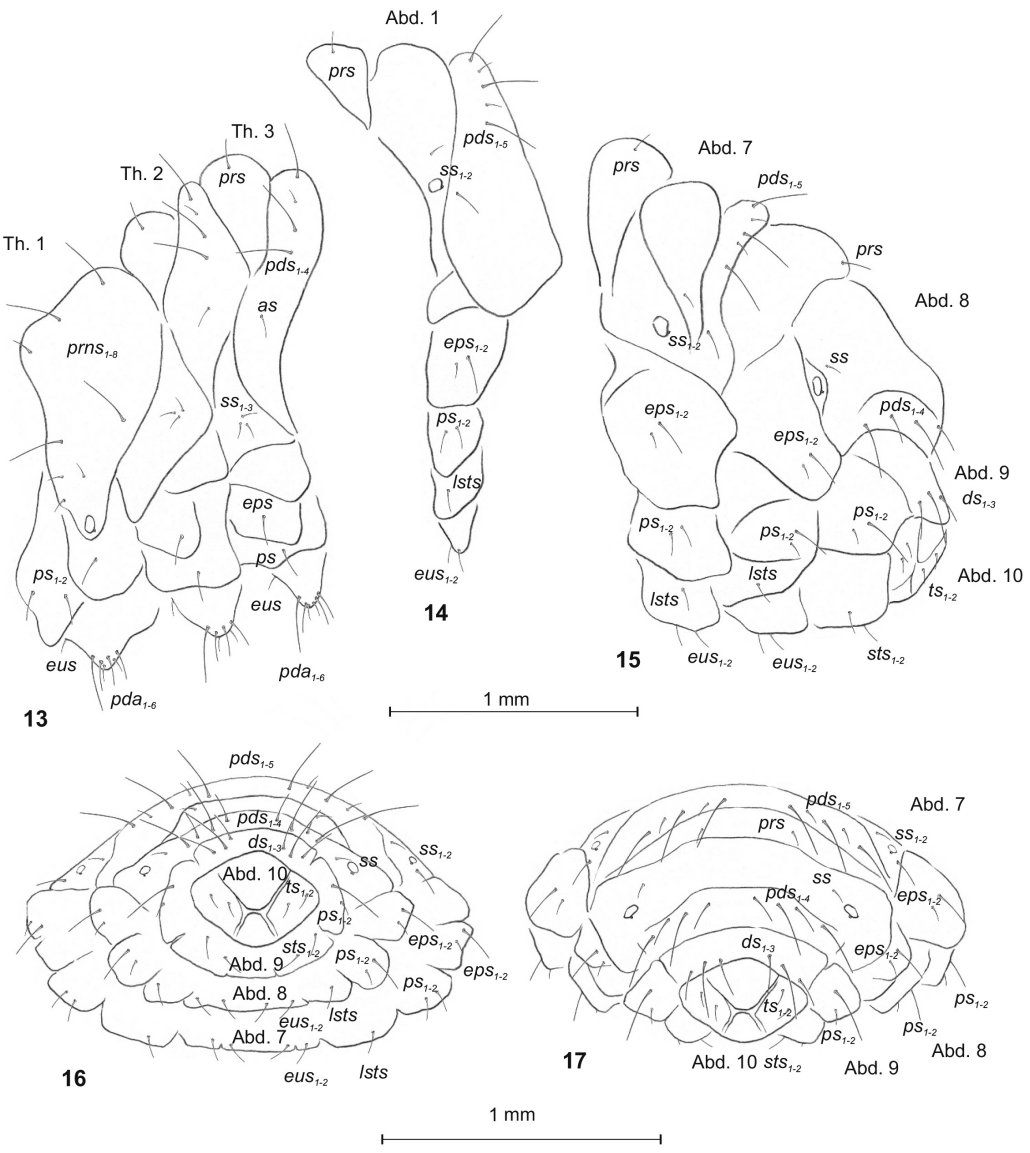
*Graptustriguttatustriguttatus* mature larva, habitus and chaetotaxy. **13** Thoracic segments, lateral view **14** First abdominal segment lateral view **15** Abdominal segments 7–10 lateral view **16** Abdominal segments 7–10 ventral view **17** Abdominal segments 7–10 dorsal view. Abbreviations: Th. 1–3 – thoracic segments 1–3, Abd. 1–10 – abdominal segments 1–10, setae: *as* – alar, *ps* – pleural, *eps* – epipleural, *ds* – dorsal, *lsts* – laterosternal, *eus* – eusternal, *pda* – pedal, *pds* – postdorsal, *prns* – pronotal, *prs* – prodorsal, *ss* – spiracular, *sts* – sternal, *ts* – terminal.

Body (Figs 1–3). Moderately slender, curved, rounded in cross section. Prothorax slightly narrower than mesothorax; metathorax as wide as mesothorax. Abdominal segments 1–6 of almost equal length; 7–9 decreasing gradually to the terminal body part; 10 reduced to 4 anal lobes with the largest in dorsal and the smallest in ventral position, lateral lobes of equal size (Fig. 3). Spiracles (of thoracic and abdominal segments 1–8) annular with 2 vestigial airtubes. Chaetotaxy well developed, setae capilliform, variable in length, dark yellow to brown. Each side of prothorax (Fig. 13) with 8 *prns* of unequal length: 5 of them placed on the weakly visible premental sclerite, next 3 short setae close to spiracle; 2 *ps* and 1 *eus.* Meso- and metathorax (Fig. 13) on each side with 1 short *prs*, 4 *pds*, variable in length: first, third and fourth long, second very short, 1 short *as*, 3 short *ss*, 1 moderately long *eps*, 1 moderately long *ps* and 1 *eus.* Each pedal area of thoracic segments with 6 *pda*, variable in length. Abd. 1–7 (Figs 14–17) on each side with 1 short *prs*, 5 *pds* variable in length(first, third and fifth long, second and fourth short) and arranged along the posterior margin of each segment, 1 minute and 1 short *ss*, 2 *eps* and 2 *ps* of various length, 1 *lsts* and 2 short *eus.* Abd. 8 (Figs 15–17) on each side with 1 short *prs*, 4 *pds* variable in length (first and third moderately long, second and fourth long) and arranged along the posterior margin of the segment, 1 minute *ss*, 2 *eps* and 2 *ps* of various length, 1 *lsts* and 2 short *eus.* Abd. 9 (Figs 15–17) on each side with 3 *ds* (dorsal setae), first moderately long, second and third long, all located close to the posterior margin of the segment, 1 long and 1 minute *ps* and 2 short *sts.* Each lateral anal lobe (Abd. 10) with a pair of minute setae.

**Figure 18.**
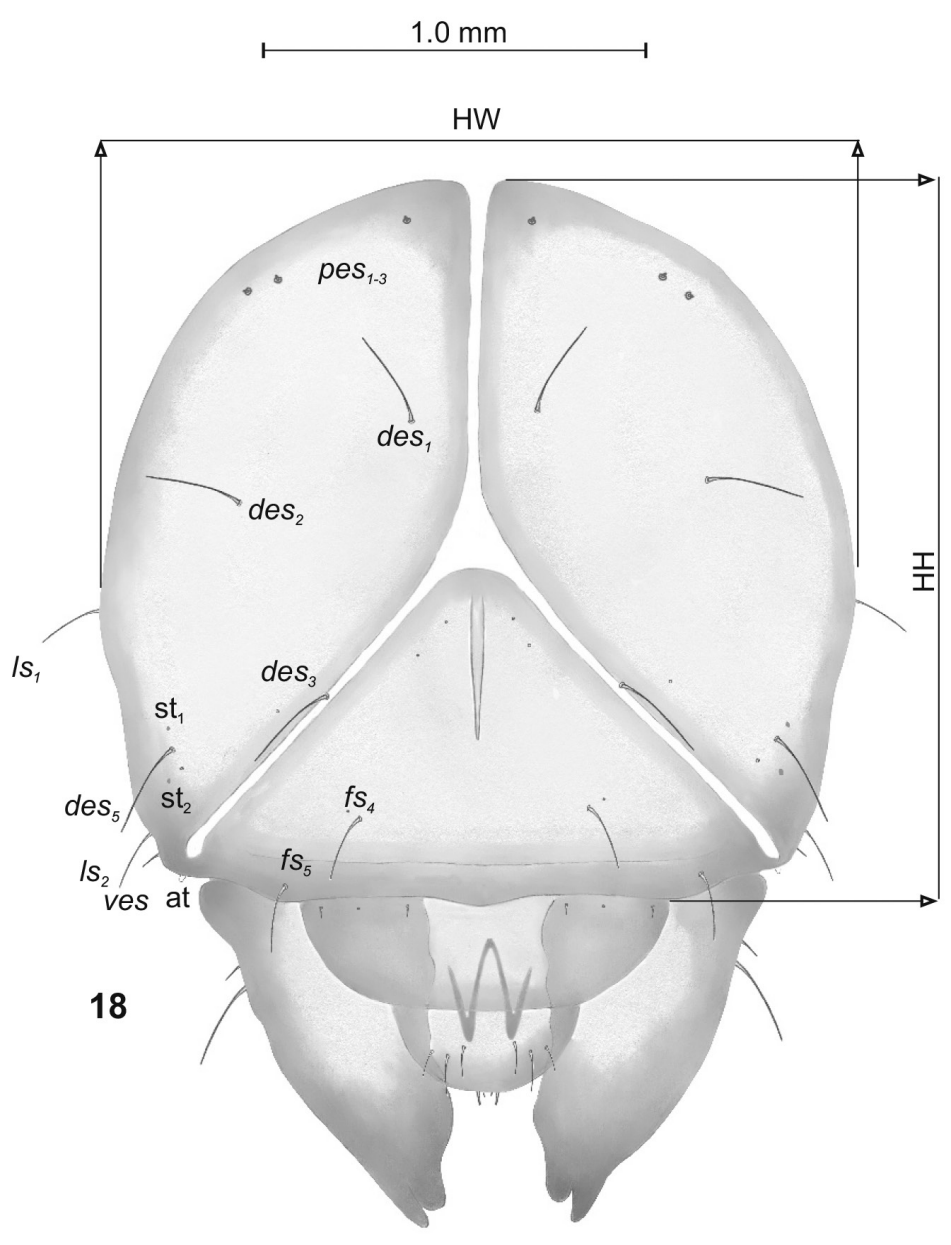
*Graptustriguttatustriguttatus* mature larva, head, frontal view. Abbreviations: at – antenna, HW – head width, HH – head height, st – stemmata, setae: *des* – dorsal epicranial, *fs* – frontal, *les* – lateral epicranial, *pes* – postepicranial, *ves* – ventral.

**Figure 19.**
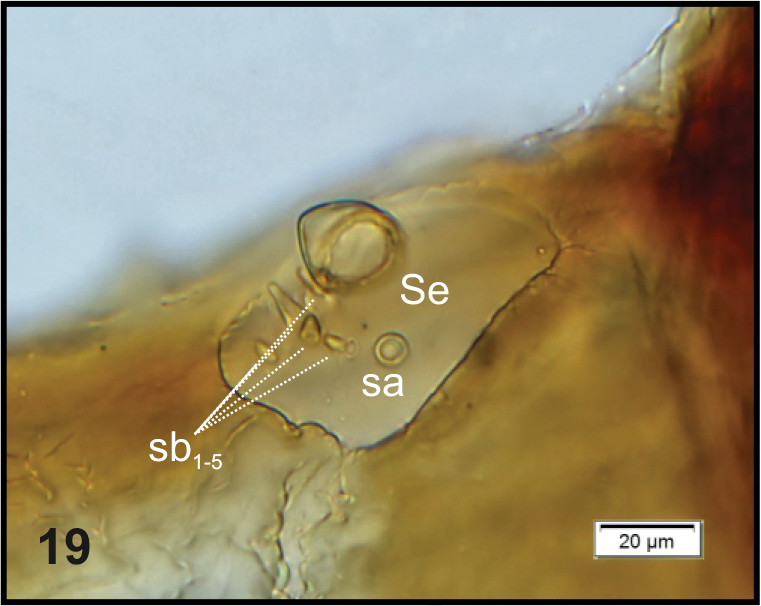
*Graptustriguttatustriguttatus* mature larva, right antenna. Abbreviations: Se – sensorium, sa – sensillum ampullaceum, sb – sensillum basiconicum.

**Figure 20.**
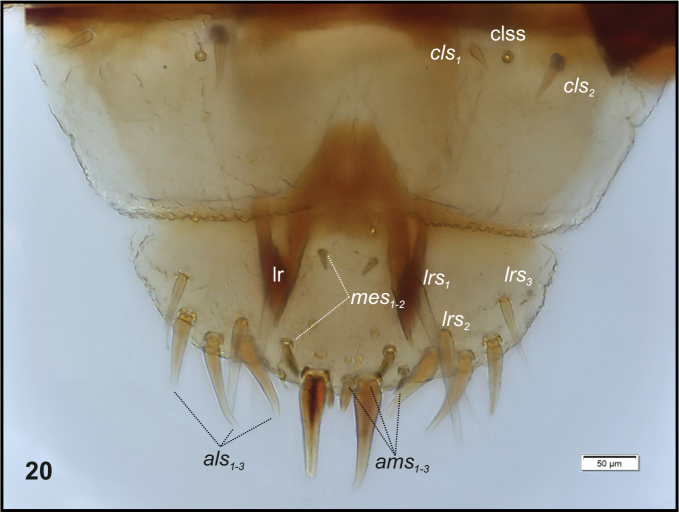
*Graptustriguttatustriguttatus* mature larva, clypeus, labrum and epipharynx. Abbreviations: clss – clypeal sensorium, lr – labral rods, setae: *als* – anterolateral, *ams* – anteromedial, *cls* – clypeal, *lrs* – labral, *mes* – median.

**Figure 21.**
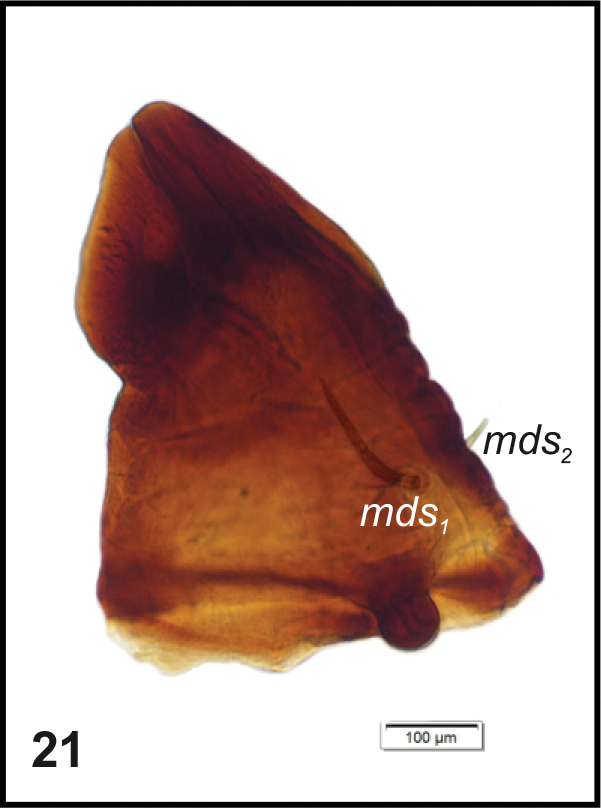
*Graptustriguttatustriguttatus* mature larva, right mandible. Abbreviations: *mds* – mandibular seta.

**Figures 22–24.**
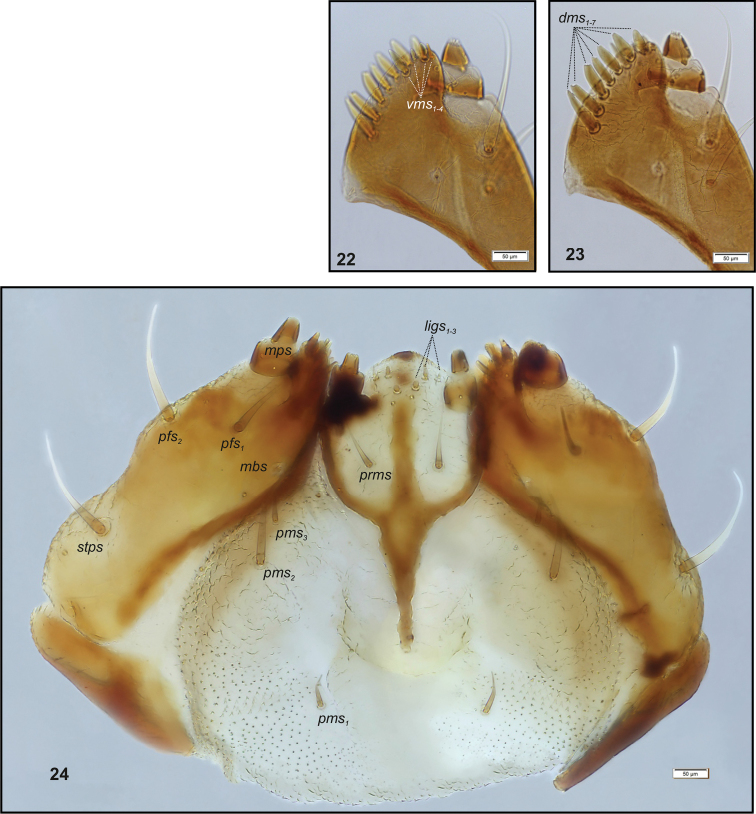
*Graptustriguttatustriguttatus* mature larva, body parts. **22** Right maxilla apical part ventral aspect **23** Right maxilla apical part dorsal aspect **24** Maxillolabial complex ventral aspect. Abbreviations: *dms* – dorsal malar, *ligs* – ligular, *mbs* – malar basiventral, *mps* – maxillary palp, *pfs* – palpiferal, *prms* – prelabial, *pms* – postlabial, *stps* – stipal, *vms* – ventral malar.

Head (Fig. 18). Light to dark yellow, oval, frontal suture distinct, Y-shaped, endocarina present, reaching to middle of frons. Setae on head capilliform; *des_1, 2, 3, 5_* equal in length; *des_1_* and *des_2_* located in the central part of epicranium, *des_3_* placed on frontal suture, *des_5_* located anterolaterally; *fs_4, 5_* equal in length, *fs_4_* located anteromedially, *fs_5_* anterolaterally, close to epistome; *les_1_* and *les_2_* equal in length, less than half the length of *des_1_*; *ves* short, poorly developed. Postepicranial area with 3 very short *pes* (Fig. 18). Two weakly visible stemmata close to *des_5_*. Antennae (Fig. 19) located at the end of frontal suture; antennal segment membranous, bearing sensorium (Se) conical, almost as wide as long, located medially, and 6 sensilla of different types: 1 sa and 5 sb. Labrum (Fig. 20) almost semicircular, anterior margin rounded; 3 pairs of *lrs*, different in length, *lrs_1_* and *lrs_2_* very long, *lrs_3_* moderately long; *lrs_1_* placed medially, *lrs_2_* anteromedially, *lrs_3_* anterolaterally. Clypeus (Fig. 20) trapezoid, its anterior margin slightly concave, covered with asperities; 2 pairs of *cls* short, located posteromedially; clss clearly visible, placed medially between *cls.* Epipharynx (Fig. 20) with 3 pairs of finger-shaped *als* of almost equal length; 3 pairs of *ams*: *ams_1_* and *ams_3_* rod-shaped, very short, *ams_2_* finger-like, very long; 2 pairs of rod-shaped *mes* of various lengths: first pair placed medially, second pair anteriorly, very close to *ams.* Surface of epipharynx smooth. Labral rods elongate, converging posteriorly. Mandibles (Fig. 21) curved, narrow, with slightly divided apex (teeth of various lengths). There is an elongate protuberance on the cutting edge between the apex and the middle of the mandible; both *mds* capilliform, different in length, placed transversely. Maxilla (Figs 22–24) with 1 *stps* and 2 *pfs* of equal length; mala with 7 finger- or rod-like *dms* of almost equal size, 4 *vms* , varied in length and all shorter than *dms*; *mbs* short. Maxillary palpi with 2 palpomeres, basal with short *mps*; distal palpomere apically with a group of sensilla, each palpomere with a pore. Basal palpomere distinctly wider than distal, both of almost equal length. Prelabium (Fig. 24) cup-like with 1 moderately long *prms*, located medially. Ligula with 3 pairs of minute *ligs*. Premental sclerite clearly visible, trident-shaped, posterior extension with acute apex. Labial palpi 2-segmented; apex of distal palpomere with some sensilla; each palpomere with a pore. Basal palpomere distinctly wider than distal, both of almost equal length. Postlabium (Fig. 24) with 3 capilliform *pms* (postlabial setae), the first pair located anteromedially, the remaining 2 pairs posterolaterally; *pms_1_* and *pms_3_* very short, *pms_2_* twice as long as others.

#### Description of the pupa.

Body length (♂, ♀): 7.5–9.0 mm; body width (at level of mesocoxae): 3.8–4.5 mm; width of thorax: 2.0–2.3 mm.

**Figures 25–27.**
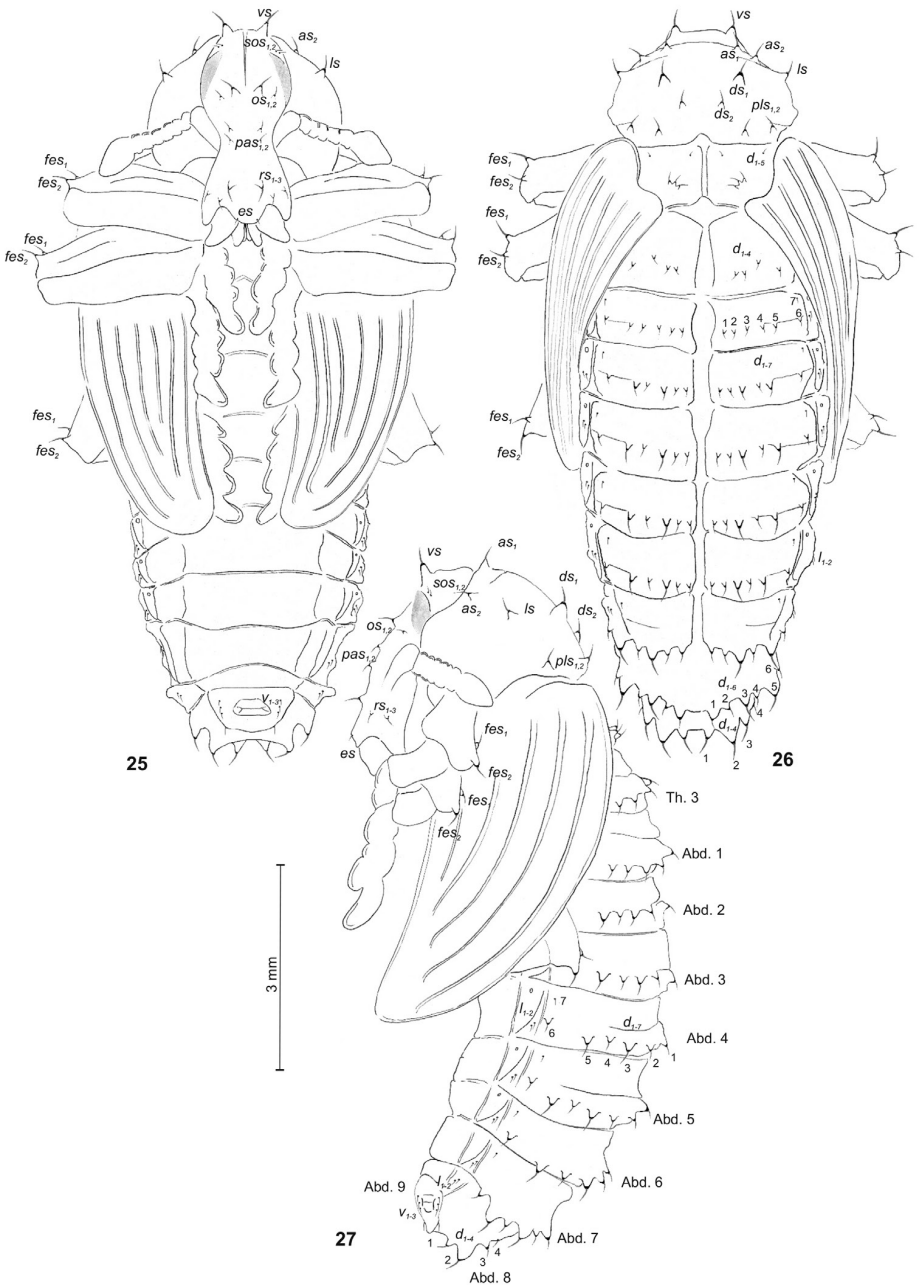
*Graptustriguttatustriguttatus* pupa. **25** Ventral **26** Dorsal **27** Lateral view. Abbreviations: Th. 1–3 – pro–, meso– and metathorax, Abd. 1–9 – abdominal segments 1–9, ur – urogomphus, setae: *as* – apical, *d* – dorsal, *ds* – discal, *es* – epistomal, *fes* – femoral, *l, ls* – lateral, *os* – orbital, *pas* – postantennal, *pls* – posterolateral, *rs* – rostral, *sos* – superorbital, *v* – ventral, *vs* – vertical.

Body moderately slender, straight, yellowish. Cuticle smooth (Figs 25–27). Rostrum short, 1.2 times as long as wide, extended beyond procoxae. Antennae moderately long and slender. Pronotum almost 1.8 times as wide as long. Abdominal segments 1–4 of almost equal length, segments 5–7 decreasing gradually, 8 semicircular, 9 distinctly smaller than previous segments. Urogomphi absent (Figs 25–27).

Chaetotaxy well developed, setae variable in lengthand shape: spine-like or capilliform, dark yellow to brown, usually located on visible protuberances. Head capsule and rostrum include 1 *vs*, 2 minute *sos*, 1 spine-like and 1 minute *os*, 2 *pas*, 3 *rs* of varied sizes and 1 minute *es.* Except *sos* and *es*, all setae of the head and rostrum are placed on protuberances. Pronotum with 2 *as*, 1 *ls*, 2 *ds* and 2 *pls.* All setae of pronotum spine-like, of equal size (only *ds_1_* slightly larger than others); all setae placed on protuberances. Mesothorax with 2 minute setae placed anteromedially and 3 spine-like setae placed medially. Metathorax with 4 spine-like setae placed medially. Abdominal segments 1–7 with 7 pairs of *d_1_*–*_7_*: *d_1_*–*_6_* short, spine-like, placed on protuberances, in lines along the posterior margin of segments, *d_7_* short, capilliform, placed anterolaterally, and 2 minute *l_1–2_*. Setae no. 3 and no. 5 increasing gradually from segment 2 to 7. Segment 8 with 4 pairs of spine-like setae of varied lengths (*d_1–4_*), placed on protuberances, in lines along the posterior margin of the segment. Seta no. 2 distinctly larger than others. Segment 9 with 3 pairs of short, capilliform *v_1–3_*. Each apex of femora with 2 *fes*, spine-like and of various length.

### Peritelus sphaeroides

Taxon classificationAnimaliaColeopteraCurculionidae

#### Specimens examined.

Rearing was started on 02.05.2012 in the climate chamber of JKI in flowerpots with mainly *Euonymusfortunei* (Turcz.) Hand.–Mazz. and one with *Prunuslaurocerasus* L. Adults had been collected 5 days previously in a hedgerow with ornamental shrubs in the JKI area.

**3 premature larvae**: flowerpot with *Euonymusfortunei*, climate chamber, JKI, 13.12.2012: 2 ex. These specimens were bred to produce pupae and transferred to Hannover for regular pupal control. As there was no further development, they were taken out on 25.01.2013; flowerpot with *Prunuslaurocerasus*, climate chamber of JKI, 14.03.2013: 1 ex.

**12 mature larvae**: flowerpot with *Euonymusfortunei*, JKI, climate chamber, 24.08.2012: 1 ex. (the first mature larva after 3 months and 3 weeks of development), 01.11.2012: 2 ex., do., 13.12.2012: 5 ex. (4 of them were used for regular pupae control; as there was no pupation, they were taken out on 25.01.2013), 14.03.2013: 2 ex., flowerpot with *Prunuslaurocerasus*, JKI, climate chamber, 14.03.2013: 2 ex.

#### Description of mature larva.

Body length: 6.5–7.7 mm, body width at the widest part (level of first abdominal segment): 2.0–2.5 mm, head width: 1.10–1.17 mm, head height: 0.90–1.00 mm.

**Figures 28–32.**
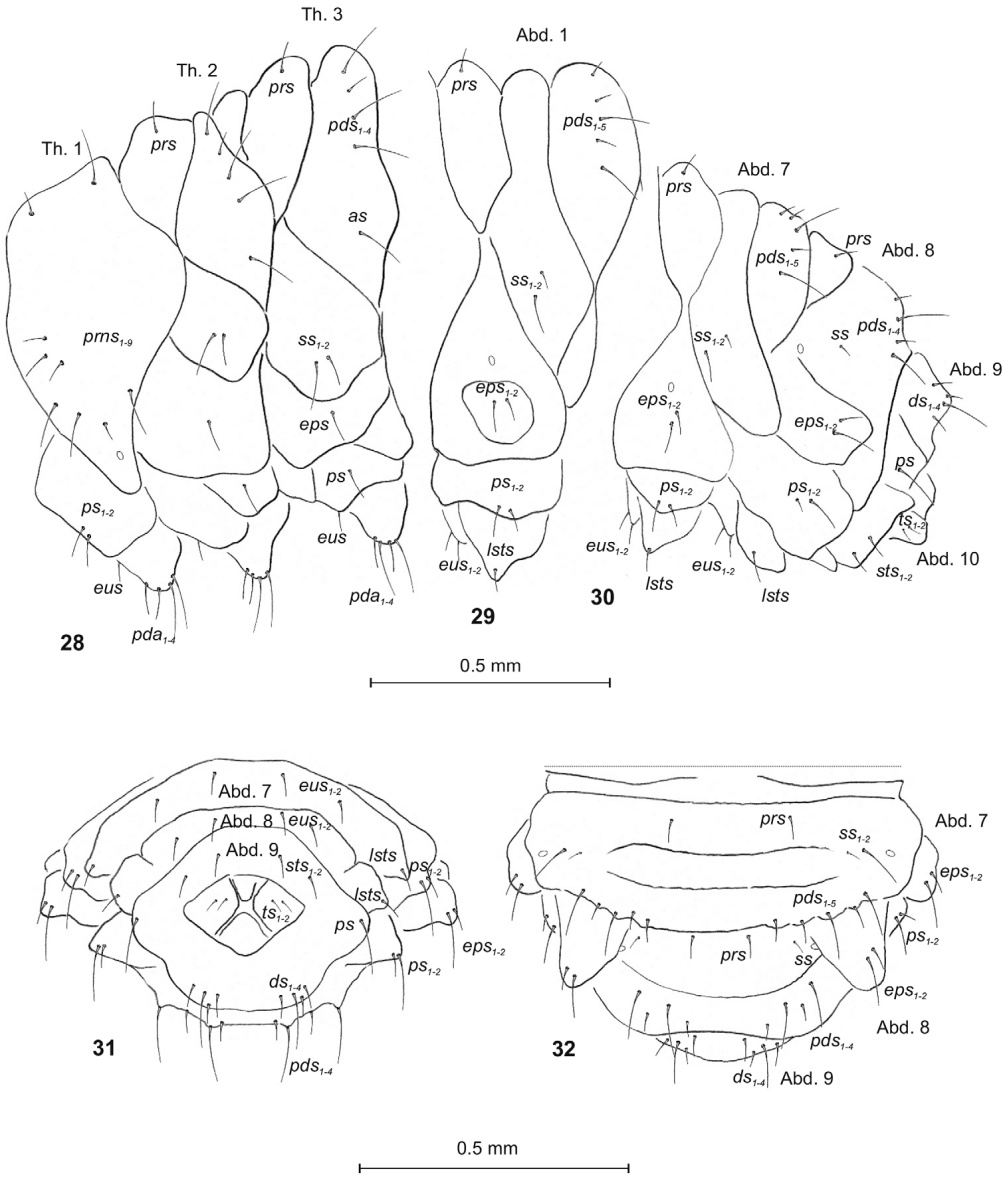
*Peritelussphaeroides* mature larva, habitus and chaetotaxy. **28** Thoracic segments lateral view **29** First abdominal segment lateral view **30** Abdominal segments 7–10 lateral view **31** Abdominal segments 7–10 ventral view **32** Abdominal segments 7–10 dorsal view Abbreviations: Th. 1–3 – thoracic segments 1–3, Abd. 1–10 – abdominal segments 1–10, setae: *as* – alar, *ps* – pleural, *eps* – epipleural, *ds* – dorsal, *lsts* – laterosternal, *eus* – eusternal, *pda* – pedal, *pds* – postdorsal, *prns* – pronotal, *prs* – prodorsal, *sps* – spiracular, *sts* – sternal, *ts* – terminal.

Body (Figs 4–6). Slender, curved, rounded in cross section. Prothorax slightly smaller than mesothorax; metathorax as wide as mesothorax. Abdominal segments 1–6 of almost equal length; 7–9 decreasing gradually to the terminal parts of the body; 10 reduced to 4 anal lobes of various sizes (ventral lobe the smallest, dorsal the largest) (Fig. 6). Spiracles (of thoracic and abdominal segments 1–8) annular. Chaetotaxy well developed; setae capilliform, variable in length, dark yellow to brown. Each side of prothorax (Fig. 28) with 9 *prns* of different length; 2 *ps* and 1 *eus.* Meso- and metathorax (Fig. 28) on each side with 1 moderately long *prs*, 4 *pds*, variable in length (first, third and fourth long, second short), 1 long *as*, 2 moderately long *ss*, 1 moderately long *eps*, 1 moderately long *ps* and 1 *eus.* Each pedal area of thoracic segments with 4 *pda*, variable in length. Abd. 1–7 (Fig. 27) on each side with 1 short *prs*, 5 *pds*, varied in length (first, second and fourth very short, third and fifth long) and arranged along the posterior margin of each segment, 1 minute and 1 long *ss*, 2 *eps* and 2 *ps* of varied lengths, 1 *lsts* and 2 short *eus.* Abd. 8 (Figs 30–32) on each side with 1 short *prs*, 4 *pds* of varied length (first and third moderately long, second and fourth long) and all arranged along posterior margin of the segment, 1 minute *ss*, 2 *eps* and 2 *ps* of various length, 1 *lsts* and 2 short *eus.* Abd. 9 (Figs 30–32) on each side with 4 *ds* (dorsal setae): first, second and fourth short, third moderately long, all located close to posterior margin of the segment, 1 long *ps* and 2 short *sts.* Each lateral anal lobe (Abd. 10) with a pair of minute terminal setae (*ts*).

**Figure 33.**
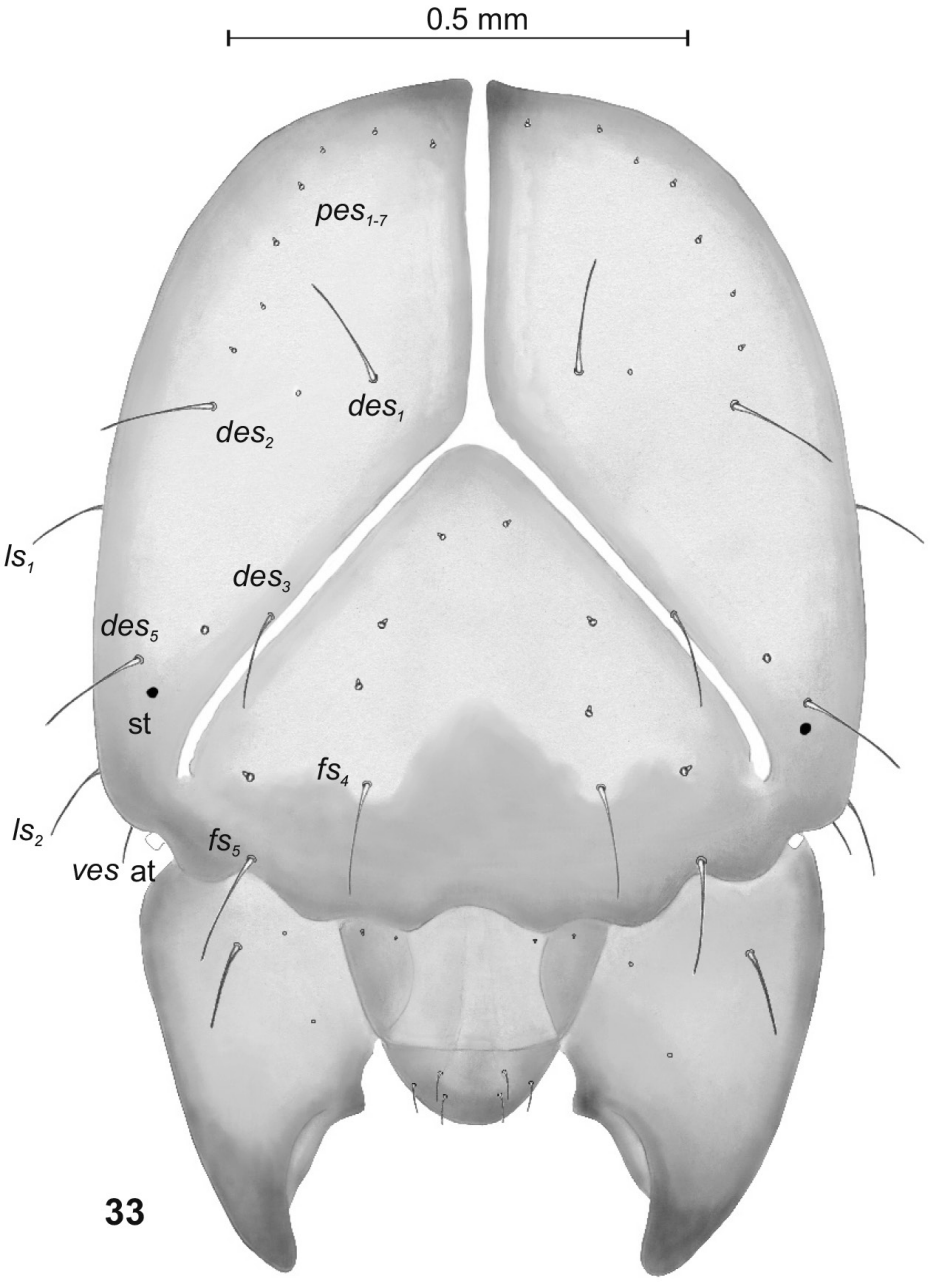
*Peritelussphaeroides* mature larva, head frontal view. Abbreviations: at – antenna, st – stemmata, setae: *des* – dorsal epicranial, *fs* – frontal, *les* – lateral epicranial, *pes* – postepicranial, *ves* – ventral.

**Figure 34.**
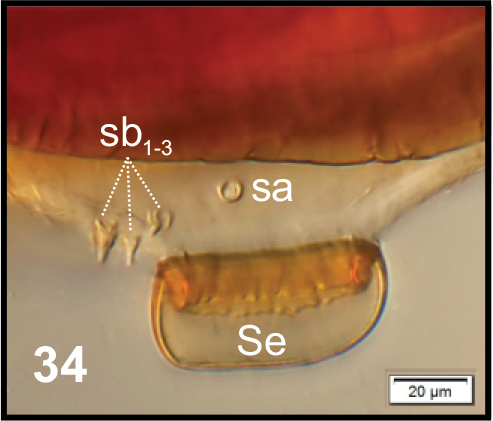
*Peritelussphaeroides* mature larva, right antenna. Abbreviations: Se – sensorium, sa – sensillum ampullaceum, sb – sensillum basiconicum.

**Figures 35–38.**
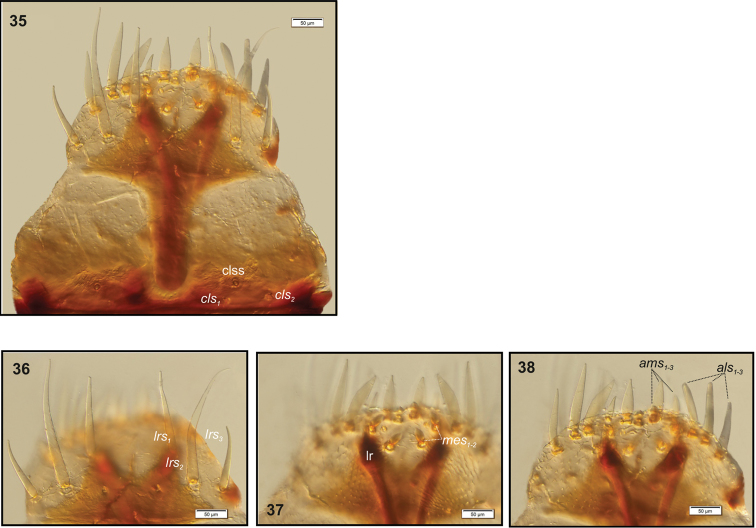
*Peritelussphaeroides* mature larva, body parts. **35** Clypeus and labrum **36** Clypeus **37, 38** Epipharynx. Abbreviations: clss – clypeal sensorium, lr – labral rods, setae: *als* – anterolateral, *ams* – anteromedial, *cls* – clypeal, *lrs* – labral, *mes* – median.

**Figures 39, 40.**
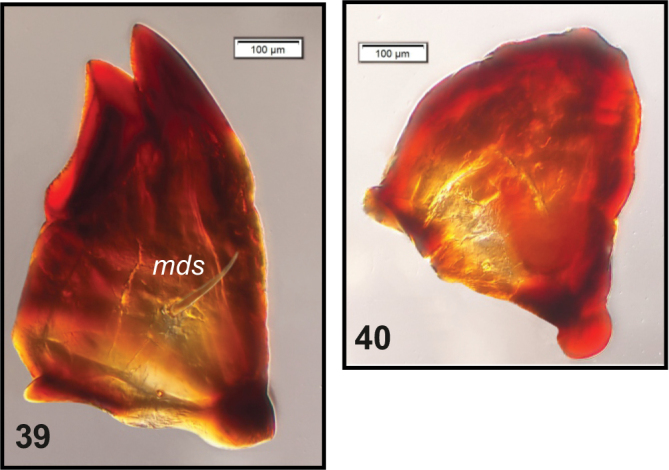
*Peritelussphaeroides* mature larva, right mandible. **39** Typical **40** Worn out. Abbreviations: *mds* – mandibular seta.

**Figures 41–43.**
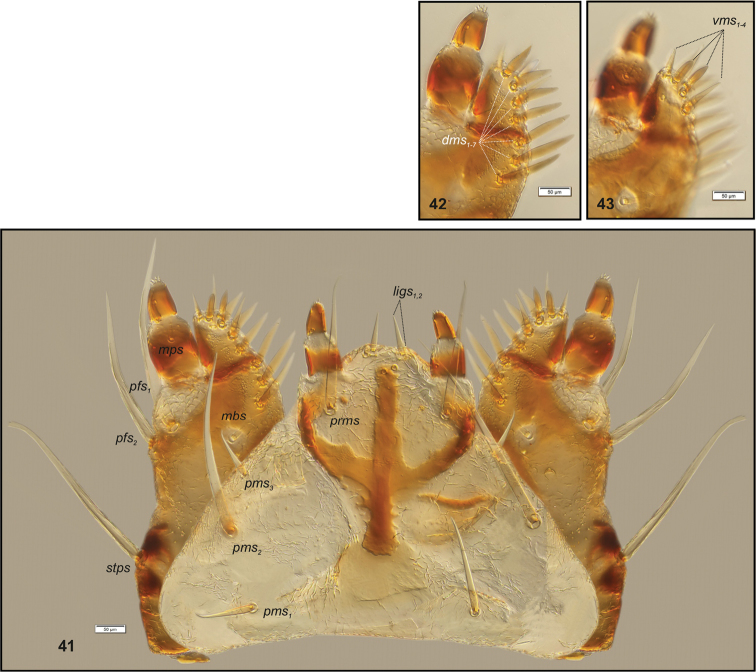
*Peritelussphaeroides* mature larva, body parts. **41** Maxillolabial complex ventral aspect **42** Right maxilla apical part dorsal aspect **43** Right maxilla apical part ventral aspect. Abbreviations: setae: *dms* – dorsal malar, *ligs* – ligular, *mbs* – malar basiventral, *mps* – maxillary palp, *pfs* – palpiferal, *prms* – prelabial, *pms* – postlabial, *stps* – stipal, *vms* – ventral malar.

Head (Fig. 33). Light to dark yellow, slightly narrowed bilaterally, frontal suture distinct, Y-shaped, endocarina absent. Setae on head capilliform; *des_1, 2, 3, 5_* equal in length; *des_1_* and *des_2_* located in the central part of epicranium, *des_3_* placed on frontal suture, *des_5_* located anterolaterally; *fs_4, 5_* almost equal in length, *fs_4_* located anteromedially, *fs_5_* anterolaterally, close to epistome; *les_1_* and *les_2_* equal in length, slightly shorter than *des_1_*. Postepicranial area with 7 very short *pes.* Single *ves* very short (Fig. 33). Stemmata poorly visible, located close to *des_5_*. Antenna (Fig. 34) located at the end of frontal suture; antennal segment membranous, bearing cushion-like sensorium (Se), located medially and 4 sensilla of different types: 1 ampullaceum (sa) and 3 basiconica (sb). Clypeus (Fig. 35) trapezoid, anterior margin of clypeus slightly emarginate at the inside; 2 pairs of *cls* very short, located posteromedially; *clss* clearly visible, placed medially between *cls.* Labrum (Figs 35, 36) almost semicircular, anterior margin rounded; 3 pairs of *lrs* of different length, *lrs_1_* and *lrs_3_* moderately long, *lrs_2_* very long, all *lrs* reaching behind anterior margin of labrum; *lrs_1_* placed medially, *lrs_2_* anteromedially, *lrs_3_* anterolaterally. Epipharynx (Figs 37, 38) with 3 pairs of finger-shaped *als* of almost equal length; 3 pairs of *ams*: *ams_1_* and *ams_3_* rod-shaped, very short, *ams_2_* finger-like, very long; 2 pairs of rod-shaped *mes*, equal in length. Surface of epipharynx covered with asperities. Labral rods elongate, converging posteriorly. Mandibles (Figs 39, 40) elongate, narrow, with divided apex (teeth variable in length). There is a protruding additional tooth on the cutting edge between the apex and the middle of the mandible; single *mds* capilliform, moderately long. These characters can disappear due to intensive feeding and gradual wear and tear of mandibles (Fig. 40). Maxilla (Figs 41–43) with 1 *stps* and 2 *pfs* of equal length; mala with 7 finger-like *dms* (Fig. 42) and 4 *vms*, all of varied length, the latter only slightly shorter than *dms* (Fig. 43); *mbs* short. Maxillary palpi with 2 palpomeres, basal with short *mps*; distal palpomere apically with a group of sensilla, each palpomere with a pore. Basal palpomere distinctly wider and longer than distal. Prelabium (Fig. 41) cup-like with 1 very long *prms*, located medially. Ligula with 2 pairs of *ligs*: first relatively long, second minute. Premental sclerite clearly visible, trident-shaped, posterior extension with thickened apex. Labial palpi 2-segmented; apex of distal palpomere with some sensilla; each palpomere with a pore. Basal palpomere distinctly wider and slightly longer than distal. Postlabium (Fig. 41) with 3 capilliform *pms*, the first pair located anteromedially, the remaining 2 pairs posterolaterally: *pms_1_* moderately long, *pms_2_* twice as long as*pms_1_* and *pms_3_* short.

### Philopedon plagiatum

Taxon classificationAnimaliaColeopteraCurculionidae

#### Specimens examined.

**6 premature larvae**: Germany, Niedersachsen, Hannover-Vahrenheide, Kugelfangtrift, nutrient-poor sandy grassland, collected at sparsely grown sites under *Plantagolanceolata*, 02.10.2011: 3 ex., 11.11.2011: 3 ex.

**3 mature larvae**: same site as before, 02.10.2011: 1 ex., 11.11.2011: 1 ex.; Denmark, Syddanmark, Emmerlev Klev near Højer, moraine at the sandy sea shore of the North Sea, collected on 13.08.2015: 1 ex., between the roots of PlantagomaritimaL.subsp.maritima, very probably; *P.lanceolata* was also present nearby.

#### Description of the mature larva.

Body length: 6.5–8.2 mm, body width at the widest part (level of first abdominal segment): 2.4–3.4 mm, head width: 0.97–1.03 mm, head height: 0.75–0.83 mm.

**Figures 44–48.**
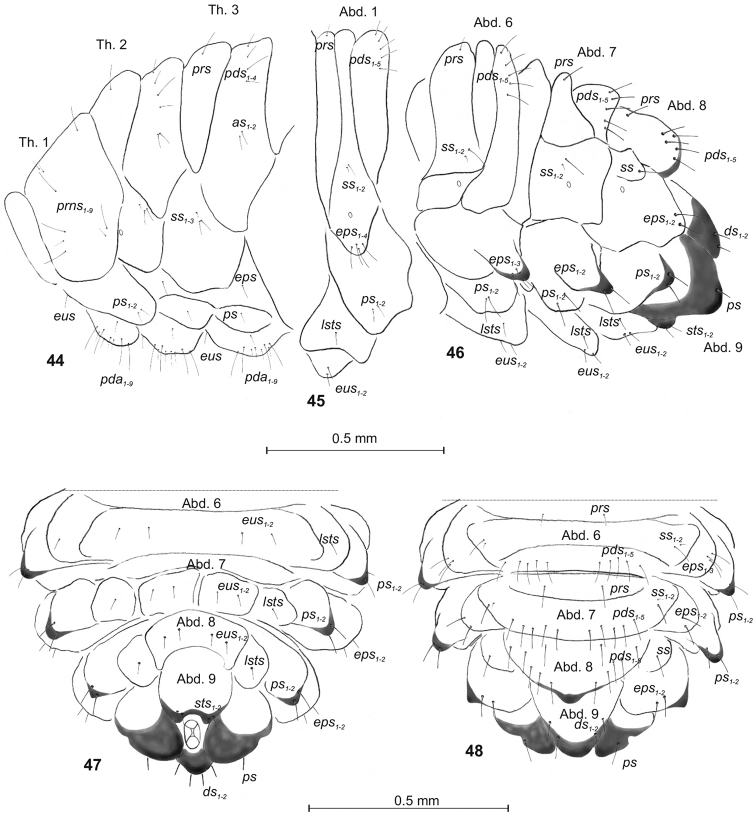
*Philopedonplagiatum* mature larva, habitus and chaetotaxy. **44** Thoracic segments lateral view **45** First abdominal segment lateral view **46** Abdominal segments 7–10 lateral view **47** Abdominal segments 7–10 ventral view **48** Abdominal segments 7–10 dorsal view. Abbreviations: Th. 1–3 – thoracic segments 1–3, Abd. 1–10 – abdominal segments 1–10, setae: *as* – alar, *ps* – pleural, *eps* – epipleural, *ds* – dorsal, *lsts* – laterosternal, *eus* – eusternal, *pda* – pedal, *pds* – postdorsal, *prns* – pronotal, *prs* – prodorsal, *sps* – spiracular, *sts* – sternal.

Body (Figs 7–9). Slender, elongate, slightly narrowed bilaterally dorso-ventrally. Prothorax slightly smaller than mesothorax; metathorax as wide as mesothorax. Abdominal segments 1–7 of almost equal length. Abdominal segment 8 wide, flattened posteriorly, with conical lateral lobes. Abdominal segment 9 strongly reduced, consisting of 4 well-isolated lobes, ventral almost rounded, lateral conical, dorsal semicircular. Abdominal segment 10 consists of 4 anal lobes of almost equal size. Anus located terminally, covered by lobes of abdominal segment 9. Apical parts of lateral lobes of the segments 6–8 and all lobes of segment 9 darkly sclerotized (Figs 46–48). Spiracles (of thoracic and abdominal segments 1–8) annular. Chaetotaxy well developed, setae capilliform, variable in length, yellowish to brown. Each side of prothorax (Fig. 44) with 9 *prns* of unequal length, placed on the weakly sclerotized pronotal sclerite; 2 *ps* and 1 *eus* very short. Meso- and metathorax (Fig. 44) on each side with 1 moderately long *prs* and 4 *pds*, variable in length (first, second and fourth short, third moderately long), 2 short *as*, 3 minute (various in length) *ss*, 1 moderately long *eps*, 1 short *ps* and 1 *eus.* Each pedal area of thoracic segments with 9 *pda*, variable in length. Abd. segment 1–8 (Figs 45–48) on each side with 1 short *prs* and 5 *pds*, almost equal in length, arranged along the posterior margin of each segment, 1 minute and 1 long *ss* (segment 8 with 1 minute *ss* only), 4 *eps* (segment 6 with 3 *eps*, segments 7 and 8 with 2 *eps*) and 2 *ps*, equal in length, 1 *lsts* and 2 short *eus.* Abdominal setae increase slightly and gradually from segment 1 to 8. Abd. segment 9 (Figs 46–48) on each side with 2 moderately long *ds*, located near the posterior margin of the segment, 1 moderately long *ps* and 2 short *sts.* Anal lobes without setae.

**Figure 49.**
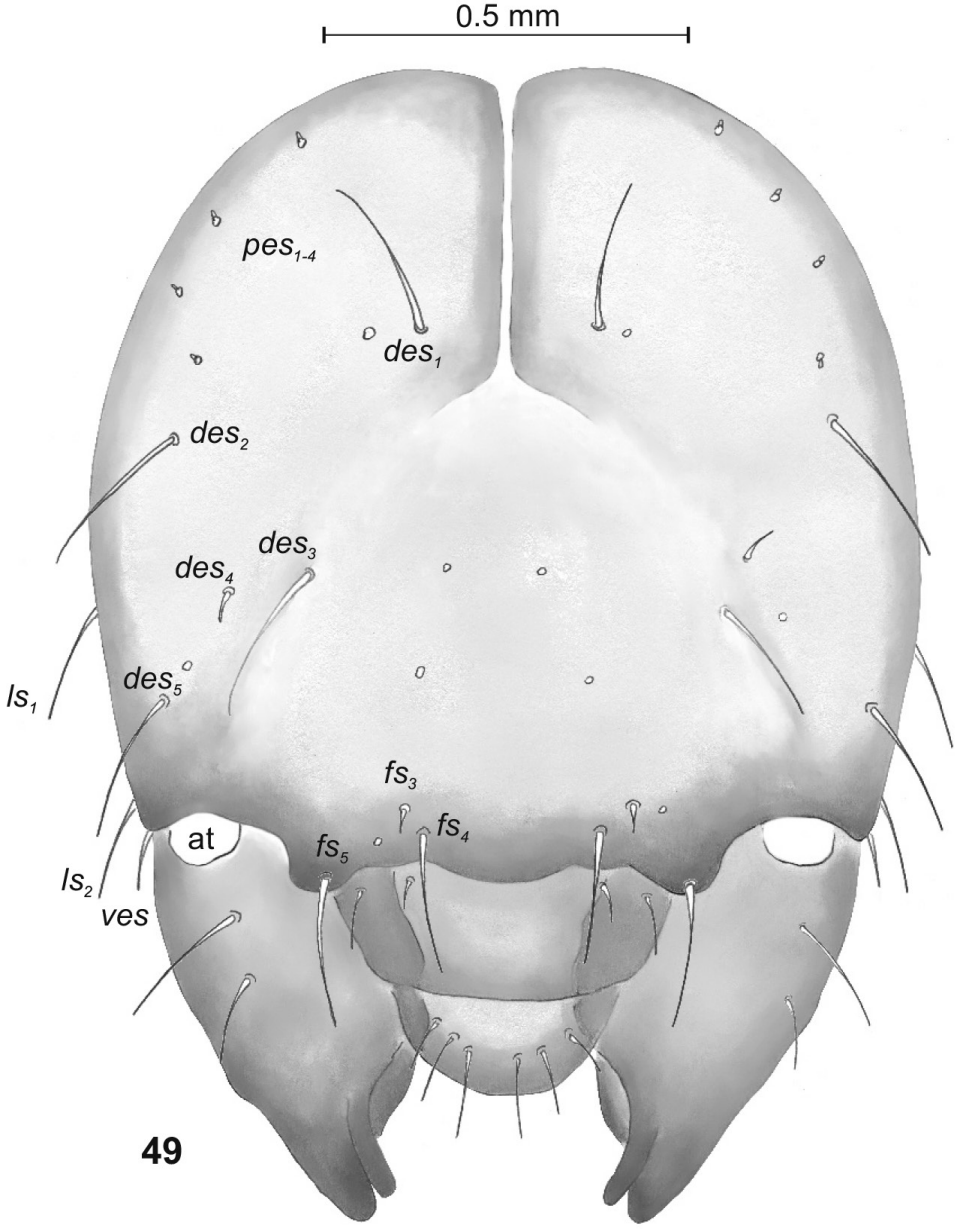
*Philopedonplagiatum* mature larva, head, frontal view. Abbreviations: at – antenna, setae: *des* – dorsal epicranial, *fs* – frontal, *les* – lateral epicranial, *pes* – postepicranial, *ves* – ventral.

**Figures 50–51.**
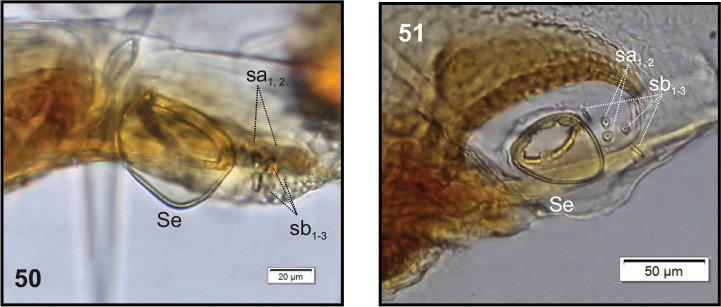
*Philopedonplagiatum* mature larva, right antenna. **50** Cushion-like **51** Conical–shaped. Abbreviations: Se – sensorium, sa – sensillum ampullaceum, sb – sensillum basiconicum.

**Figures 52–54.**
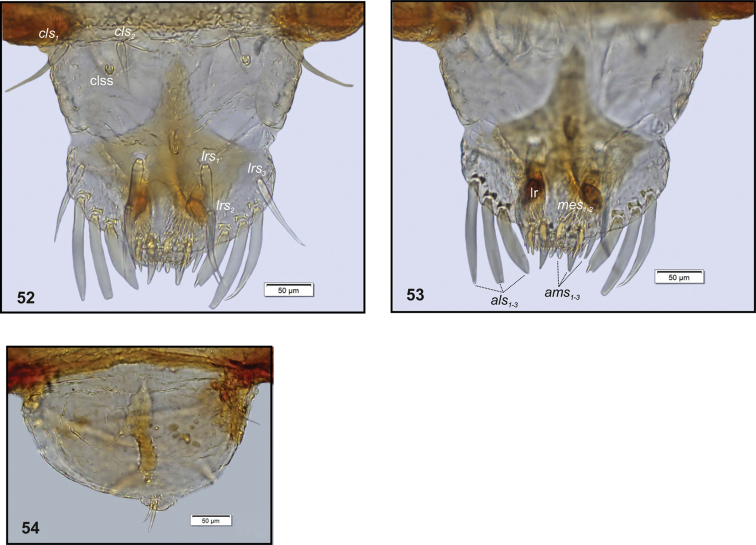
*Philopedonplagiatum* mature larva, body parts. **52** Clypeus and labrum **53** Epipharynx **54** Clypeus and labrum, deformed. Abbreviations: clss – clypeal sensorium, lr – labral rods, setae: *als* – anterolateral, *ams* – anteromedial, *cls* – clypeal, *lrs* – labral, *mes* – median.

**Figure 55.**
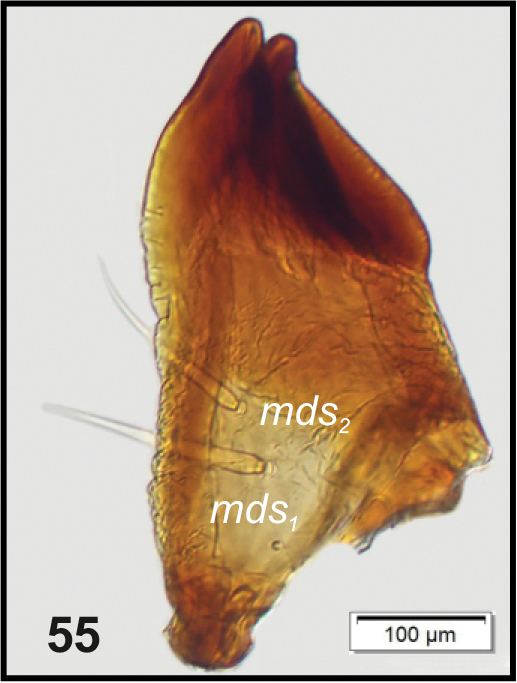
*Philopedonplagiatum* mature larva, left mandible. Abbreviations: *mds* – mandibular seta.

**Figures 56–58.**
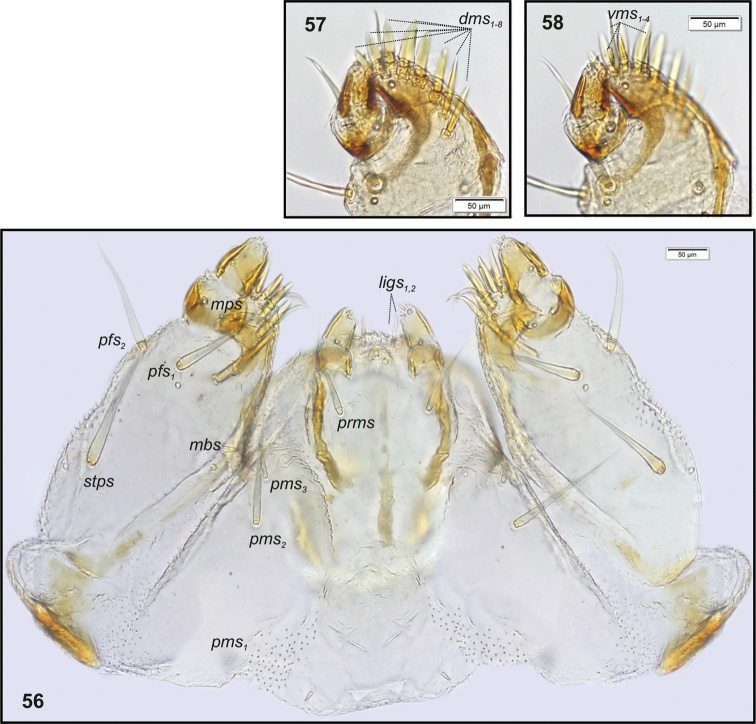
*Philopedonplagiatum* mature larva, body parts. **56** Maxillolabial complex ventral aspect **57** Right maxilla apical part dorsal aspect **58** Right maxilla apical part ventral aspect. Abbreviations: setae: *dms* – dorsal malar, *ligs* – ligular, *mbs* – malar basiventral, *mps* – maxillary palp, *pfs* – palpiferal, *prms* – prelabial, *pms* – postlabial, *stps* – stipal, *vms* – ventral malar.

Head (Fig. 49). Greyish to light yellow, suboval, slightly oblate bilaterally; frontal suture almost invisible, endocarina absent. Setae on head capilliform; *des_1, 2, 3, 5_* long, equal in length, *des_4_* very short; *des_1_* and *des_2_* located in the central part of epicranium, *des_3_* and *des_4_* placed on epicranium close to *des_3_*, *des_5_* located anterolaterally; *fs_4, 5_* equal in length, *fs_3_* very short; *fs_3_* placed medially, *fs_4_* located anteromedially, *fs_5_* anterolaterally, close to epistome; *les_1_* and *les_2_* equal in length, slightly shorter than *des_1_*. Single *ves* moderately long. Postepicranial area with 4 very short *pes* (Fig. 49). Antenna (Figs 50, 51) located at the end of frontal suture; antennal segment membranous, bearing sensorium usually cushion-like, truncate at apex (Se) (Fig. 50) or occasionally conical-like (Fig. 51), and 5 sensilla: 2 ampullacea (sa) and 3 basiconica (sb). Labrum (regular) (Figs 52, 53) (deformed) (Fig. 54) almost semicircular, anterior margin doubly sinuate; 3 pairs of *lrs* almost equal in length; *lrs_1_* placed medially, *lrs_2_* anteromedially, *lrs_3_* anterolaterally. Clypeus (Figs 52, 53) trapezoid, anterior margin of clypeus straight; 2 pairs of *cls* almost as long as*lrs*–*cls_1_* located posterolaterally, *cls_2_* posteromedially; clss clearly visible placed posteriorly, between *cls.* Epipharynx (Fig. 53) with 3 pairs of finger-shaped *als* of various lengths; 3 pairs of rod-shaped *ams*: *ams_1_* and *ams_3_* short, *ams_2_* moderately long; 2 pairs of rod-shaped *mes*, equal in length, both placed anteromedially very close to *ams.* Surface of epipharynx (especially close to margin) covered with asperities. Labral rods short, rounded, slightly converging posteriorly. Mandibles (Fig. 55) curved, narrow, with divided apex (teeth different in length). There is an elongated protuberance on the cutting edge between the apex and the middle of the mandible; both *mds* capilliform, almost equal in length. Maxilla (Figs 56–58) with 1 *stps* and 2 *pfs* of equal length; mala with 8 finger- or rod-like *dms* (Fig. 57) and 4 *vms*, both varied in length (Fig. 58); *mbs* very short. Maxillary palpi with 2 palpomeres, basal with short *mps*; distal palpomere apically with a group of sensilla, each palpomere with a pore. Basal palpomere wider than distal, both of almost equal length. Prelabium (Fig. 56) cup-like with 1 long *prms*, located medially. Ligula with 2 pairs of *ligs*, various in length. Premental sclerite clearly visible, trident-shaped (median branch weakly sclerotized), posterior extension with elongated apex. Labial palpi 2–segmented; apex of distal palpomere with some sensilla; each palpomere with a pore. Basal palpomere slightly wider and shorter than distal. Postlabium (Fig. 56) with 3 capilliform *pms*, the first pair located anteromedially, the remaining 2 pairs posterolaterally; *pms_1_* minute, *pms_2_* very long, *pms_3_* moderately long. Surface of postlabium and stipes partially covered with asperities.

### Tanymecus palliatus

Taxon classificationAnimaliaColeopteraCurculionidae

#### Specimens examined.

**3 mature larvae**: Germany, Brandenburg, Cottbus: Kittlitz, collected on 09.08.2011 in a permanent field of *Medicagosativa* L., together with a *Tanymecus* pupa and many larvae of *Otiorhynchusligustici* (Linnaeus, 1758).

**12 first instar larvae**: A female collected in the field (Kittlitz) laid eggs on 17.05.2012 in the laboratory in Hannover. Larvae, hatched from eggs on 31.05.2012, were used for this study.

#### Description of the mature larva.

Body length: 8.3–10.0 mm, body width at the widest part (level of first abdominal segment): 2.5–3.2 mm, head width: 1.6–1.8 mm, head height: 1.4–1.6 mm.

**Figures 59–63.**
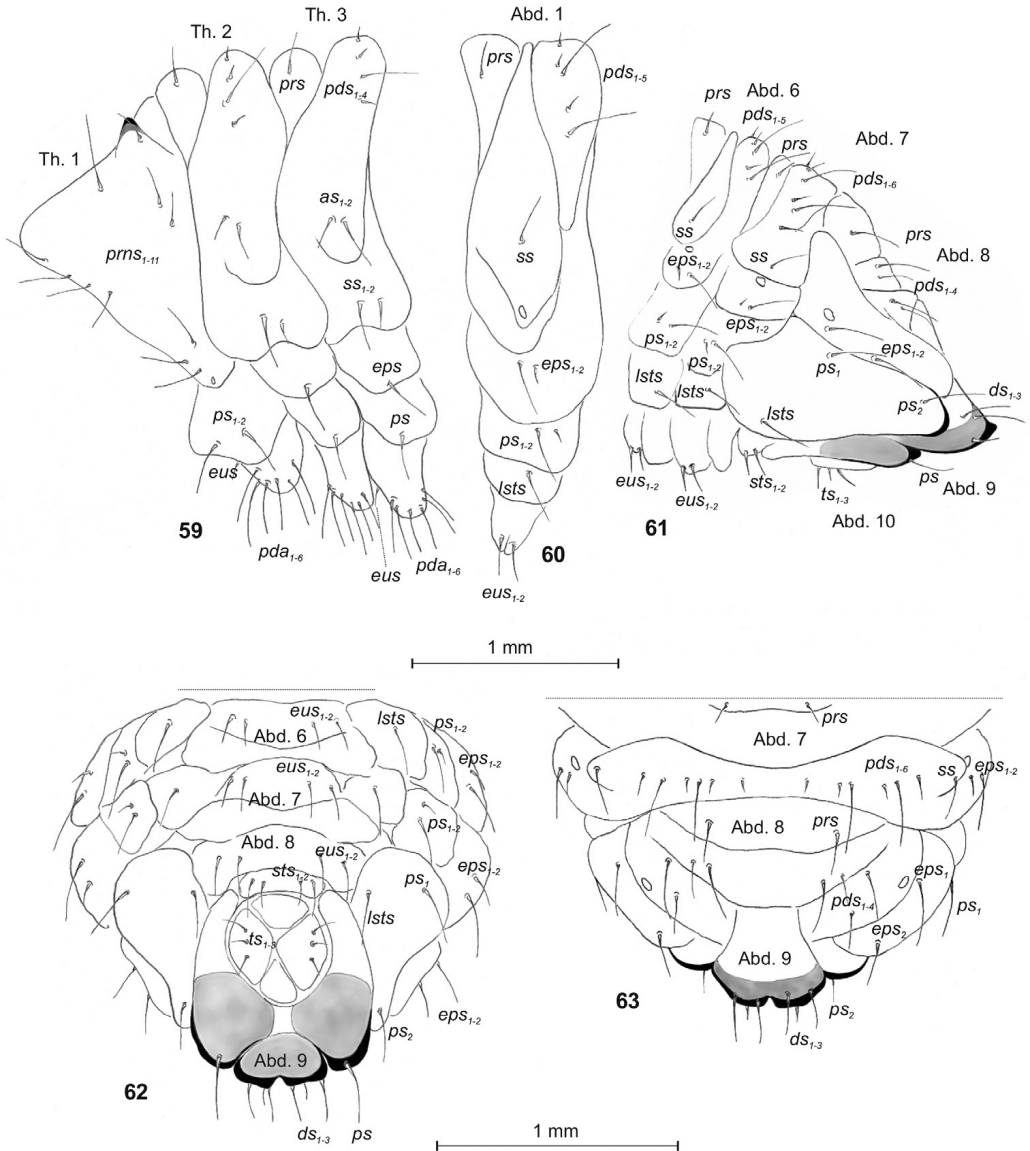
*Tanymecuspalliatus* mature larva, habitus and chaetotaxy. **59** Thoracic segments lateral view **60** First abdominal segment lateral view **61** Abdominal segments 7–10 lateral view **62** Abdominal segments 7–10 ventral view **63** Abdominal segments 7–10 dorsal view. Abbreviations: Th. 1–3 – thoracic segments 1–3, Abd. 1–10 – abdominal segments 1–10, setae: *as* – alar, *ps* – pleural, *eps* – epipleural, *ds* – dorsal, *lsts* – laterosternal, *eus* – eusternal, *pda* – pedal, *pds* – postdorsal, *prns* – pronotal, *prs* – prodorsal, *sps* – spiracular, *sts* – sternal, *ts* – terminal.

Body (Figs 10–12). Moderately stout, slightly curved, rounded in cross section. Prothorax slightly smaller than mesothorax; metathorax as wide as mesothorax. Prothorax with a pair of dark sclerotized, conical protuberances, placed dorsally, near margin with mesothorax (Fig. 59). Spiracles (of thoracic and abdominal segments 1–8) annular. Abdominal segments 1–7 of almost equal length, segment 8 wide, flattened posteriorly, with conical lateral lobes. Abdominal segment 9 strongly reduced, consisting of 4 well-isolated lobes, of which the lateral lobes are the biggest; segment 10 consists of 4 anal lobes of various size. Anus located ventrally (Figs 11, 12, 62). Chaetotaxy well developed, setae capilliform, variable in length, greyish or yellowish. Each side of prothorax (Fig. 59) with 11 *prns* of almost equal size (8 of them placed on the weakly visible premental sclerite, next 3 close to the spiracle; 2 *ps* moderately long and 1 short *eus.* Meso- and metathorax on each side with 1 moderately long *prs* and 4 *pds* of various lengths (first, second and fourth very short, third long), 1 moderately long and 1 short *as*, 1 moderately long and 1 short *ss*, 1 *eps* and *1 ps*, both moderately long, 1 short *eus.* Each pedal area of thoracic segments with 6 *pda*, almost equal in length. Abd. 1–7 (Figs 60–63) on each side with 1 moderately long *prs* and 5 *pds*, of various lengths (first, second and fourth very short, third and fifth very long; Abd. 7 with 6 *pds*), arranged along the posterior margin of each segment, 1 long *ss*, 2 *eps* and 2 *ps*, both of different lengths, 1 *lsts* and 2 moderately long *eus.* Abd. 8 (Figs 61–63) on each side with 1 moderately long *prs*, 4 relatively elongate *pds*, 2 *eps* and 2 *ps*, both of different lengths, 1 *lsts* and 2 *eus.* Abd. 9 (Figs 61–63) on each side with 3 *ds*, first and third moderately long, second short, all located close to the posterior margin of the segment, 1 medium *ps* and 2 short *sts.* Each lateral anal lobe (Abd. 10) with 3 short setae (*ts_1_*–*_3_*).

**Figure 64.**
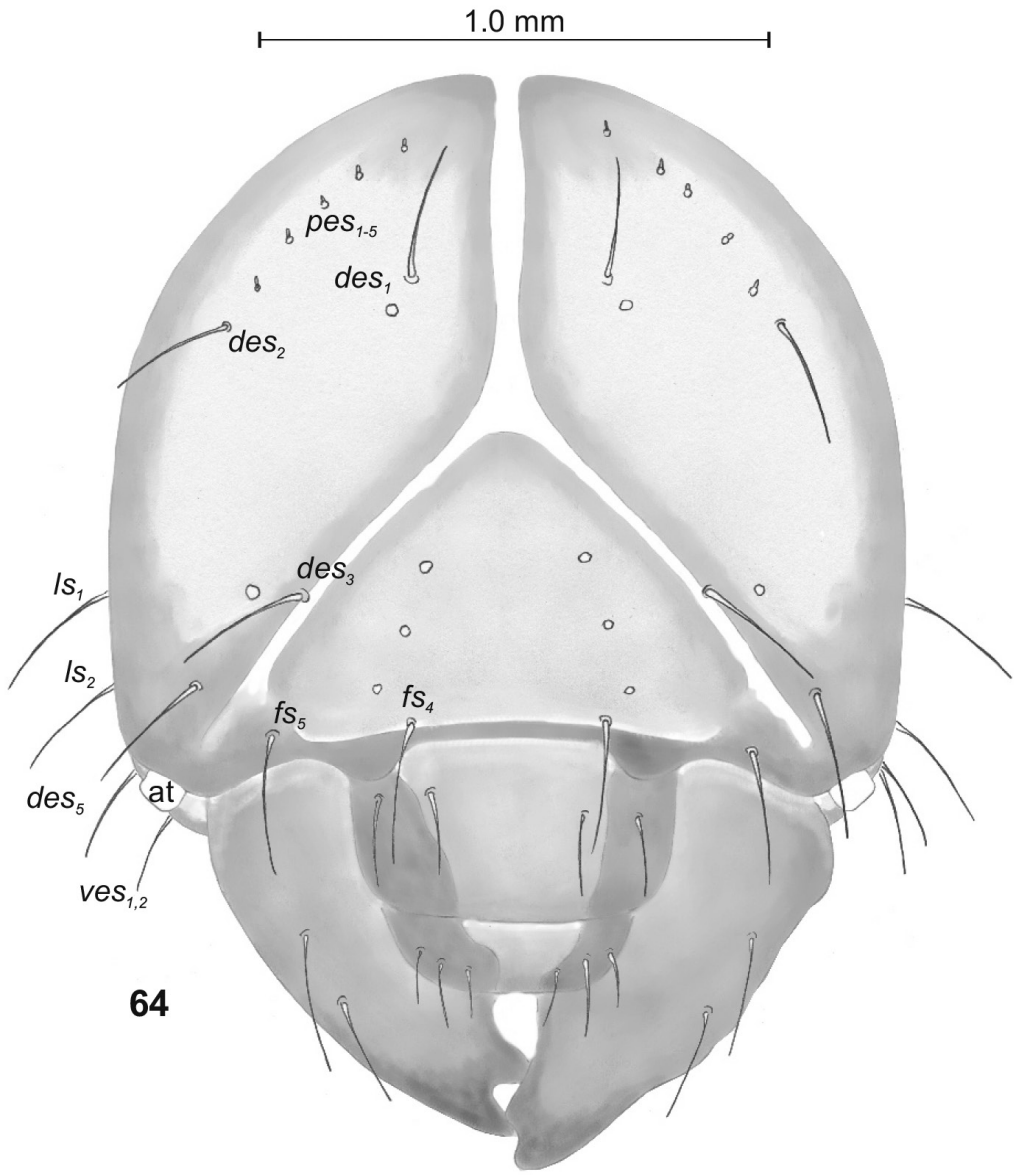
*Tanymecuspalliatus* mature larva, head, frontal view. Abbreviations: at – antenna, setae: *des* – dorsal epicranial, *fs* – frontal, *les* – lateral epicranial, *pes* – postepicranial, *ves* – ventral.

**Figure 65.**
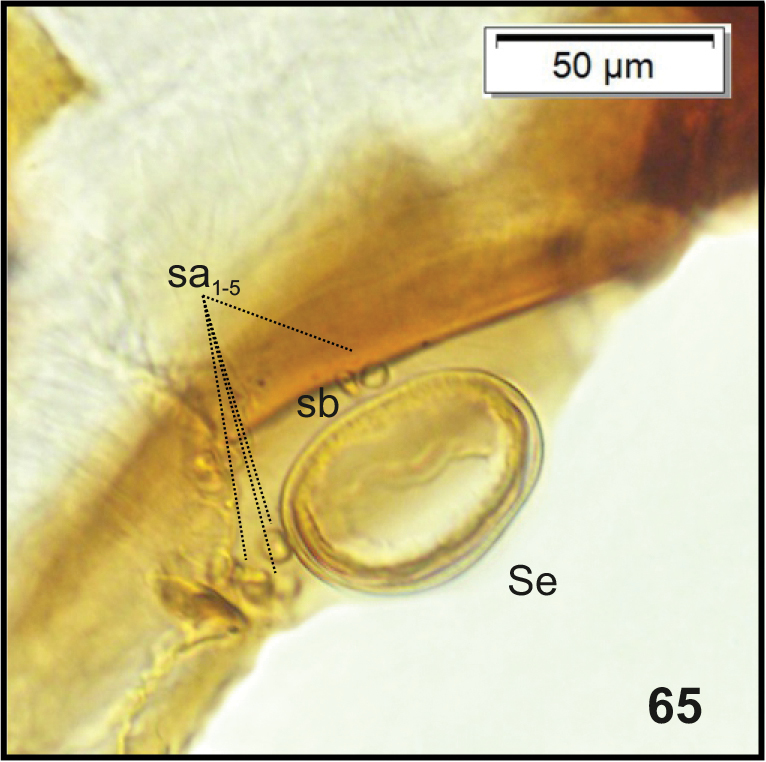
*Tanymecuspalliatus* mature larva, body parts, right antenna. Abbreviations: Se – sensorium, sa – sensillum ampullaceum, sb – sensillum basiconicum.

**Figure 66.**
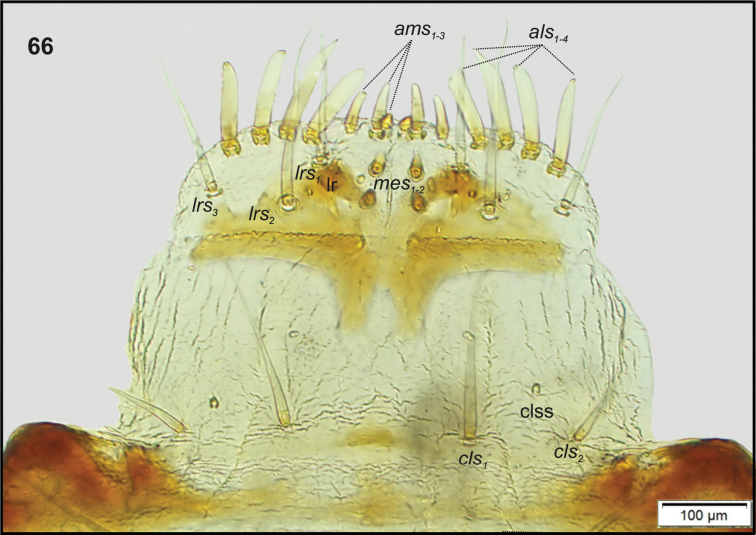
*Tanymecuspalliatus* mature larva, body parts, clypeus, labrum and epipharynx. Abbreviations: clss – clypeal sensorium, lr – labral rods, setae: *als* – anterolateral, *ams* – anteromedial, *cls* – clypeal, *lrs* – labral, *mes* – median.

**Figure 67.**
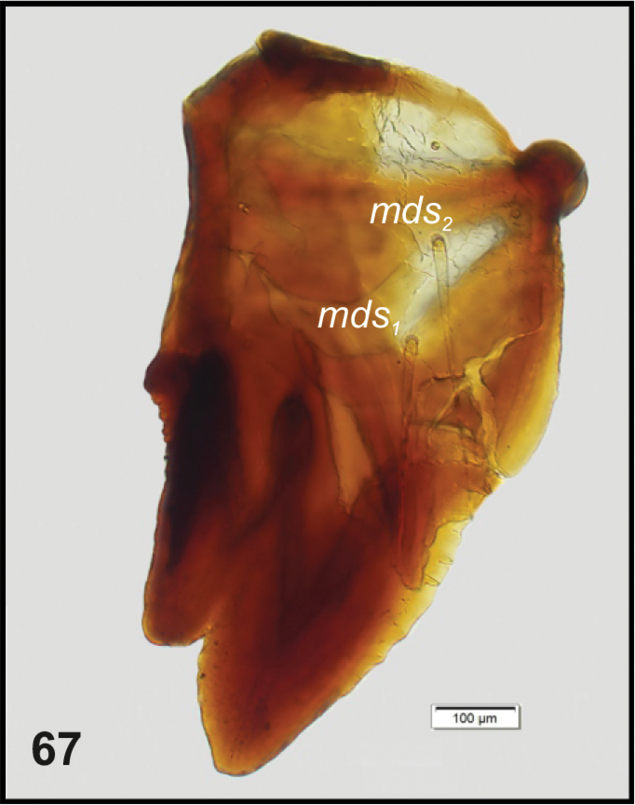
*Tanymecuspalliatus* mature larva, left mandible. Abbreviations: *mds* – mandibular seta.

**Figures 68–70.**
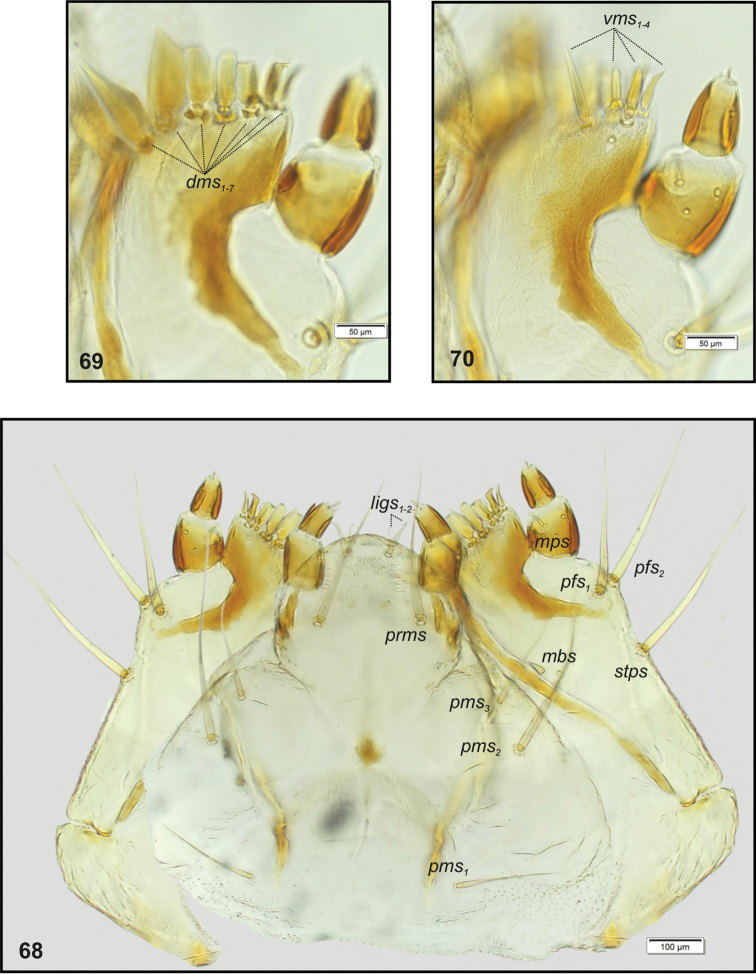
*Tanymecuspalliatus* mature larva, body parts. **68** Maxillolabial complex ventral aspect **69** Right maxilla apical part dorsal aspect **70** Right maxilla apical part ventral aspect. Abbreviations: setae: *dms* – dorsal malar, *ligs* – ligular, *mbs* – malar basiventral, *mps* – maxillary palp, *pfs* – palpiferal, *prms* – prelabial, *pms* – postlabial, *stps* – stipal, *vms* – ventral malar.

Head (Fig. 64). Greyish or light yellowish, suboval, frontal suture distinct, Y-shaped, endocarina absent. Setae on head capilliform; *des_1, 2, 3, 5_* equal in length; *des_1_* and *des_2_* located in the central part of epicranium, *des_3_* placed on frontal suture, *des_5_* located anterolaterally; *fs_4, 5_* both as long as*des_1_*, *fs_4_* located anteromedially, *fs_5_* anterolaterally, near epistome; *les_1_* and *les_2_*, equal in length, only slightly shorter than *des_1_*; *ves_1_* and *ves* almost as long as*les.* Postepicranial area with 5 very short *pes* (Fig. 64). Antenna (Fig. 65) located at the end of frontal suture; antennal segment membranous, bearing cushion-like sensorium (Se), located medially, and 6 sensilla of various types: 5 ampullacea (sa) and 1 basiconicum (sb). Labrum (Fig. 66) narrow, anterior margin slightly sinuate; 3 pairs of *lrs*, *lrs_1_* and *lrs_3_* moderately long, *lrs_2_* long; *lrs_1_* anteromedially, *lrs_2_* medially, *lrs_3_* laterally. Clypeus (Fig. 66) twice as long as than labrum, anterior margin of clypeus straight; 2 pairs of *cls*: *cls_1_*as long as*lrs_2_*, *cls_2_* less than half the length of *cls_1_*, both located posteromedially; *clss* clearly visible, placed medially between *cls.* Epipharynx (Fig. 66) with 4 pairs of finger-shaped *als* of equal length; 3 pairs of *ams*: *ams_1_* rod-shaped, very short, *ams_2_* and *ams_3_* moderately long, finger-like; 2 pairs of short, rod-shaped *mes*, both placed medially, between labral rods. Surface of epipharynx smooth. Labral rods short, converging posteriorly. Mandibles (Fig. 67) curved, narrow, with slightly divided apex (teeth of various length). There is an additional tooth on the cutting edge in the middle of the mandible; both *mds* capilliform, different in length. Maxilla (Figs 68–70) with 1 *stps* and 2 *pfs* of equal length; mala with 7 rod-like *dms* of various size (Fig. 69) and 4 capilliform *vms* variable in length(Fig. 70); *mbs* short. Maxillary palpi with 2 palpomeres, basal with short *mps*; distal palpomere apically with a group of sensilla, each palpomere with a pore. Basal palpomere distinctly wider and slightly longer than distal. Prelabium (Fig. 68) almost rounded with 1 long *prms*, located medially. Ligula with 2 pairs of *ligs* of various length. Premental sclerite clearly visible triden -shaped, median branch and posterior extension weakly sclerotized. Labial palpi 2-segmented; apex of distal palpomere with some sensilla; each palpomere with a pore. Basal palpomere wider and longer than distal. Postlabium (Fig. 68) with 3 capilliform *pms*, the first pair located anteromedially, the remaining 2 pairs posterolaterally; *pms_1_* and *pms_3_* long, *pms_2_* very long. Only posterior margin of postlabium covered with fine asperities.

##### Differentiation of described species

**Table:** 

1	Abdominal segment 9 about regular structure (type “A” Van Emden 1950)	**2**
–	Abdominal segment 9 strongly reduced, consisting of four well-isolated lobes (type “B” Van Emden 1950)	**3**
2	Abdominal segment 9 with 3 *ds*, each pedal area with 6 *pda*, meso- and metathorax with 3 *ss* each, Se conical-like	*** Graptus triguttatus triguttatus ***
–	Abdominal segment 9 with 4 *ds*, each pedal area with 4 *pda*, meso- and metathorax with 2 *ss* each, Se cushion-like	*** Peritelus sphaeroides ***
3	Abdominal segment 9 with 2 *ds*, each pedal area with 9 *pda*, meso- and metathorax with 3 *ss* each, abdominal segments 1–6 with 3–4 *eps*, clypeus almost as wide as labrum	*** Philopedon plagiatum ***
–	Abdominal segment 9 with 3 *ds*, each pedal area with 6 *pda*, meso- and metathorax with 2 *ss* each, abdominal segments 1–6 with 2 *eps*, clypeus twice as long as labrum	*** Tanymecus palliatus ***

##### The number of larval instars in *Tanymecus*

There are some strange statements about the number of larval instars in the larval stage of species of genus *Tanymecus*. Hoffmann (1963), who relied on authors from the former Soviet Union, reported about 10 larval instars in *T.palliatus*, which was already commented by Dieckmann (1983)as a ‘for weevils surprising fact’.

In *T.dilaticollis* Gyllenhal, 1834, Catrinici (1944) determined six larval instars. She reported that larval head width increased up to the fourth larval instar, decreased in the fifth and increased again in the sixth instar to nearly the same value as in the fourth. This sounds really strange and has to be taken with caution and tested with new observations. This was also the reason for Van Emden (1952) to propose four larval instars for *T.dilaticollis*.

For the exact determination of the number of larval instars we summarized and assessed our own measuring data and added data from literature, if necessary (Tables 1, 2).

Due to the dubiousness of the number of larval instars in *T.dilaticollis* given by Catrinici (1944) and Van Emden (1952) we used measuring data for the head width (HW) of adults of both species and of mature larvae of *T.palliatus* to assess the HW of the mature larva of *T.dilaticollis*. This ratio should be rather similar in two species of the same genus. Hence, the value calculated in this way for the HW of the mature larva of *T.dilaticollis* is 1.51 mm.

We also needed to determine the number of larval instars for both species: there are data for L_1_ and for mature larvae, and in *T.dilaticollis* there are also measurements for several instars, even if (especially in the higher instars) the data are doubtful.

The determination of larval instars is mainly based on the method of Dyar (1890) and has been used by several authors, even if apparently not known to all scientists who have dealt with larvae. There are several publications about weevil larvae where this ratio was applied. We preferred to use Dyar’s ratio^–1^ and called it Growth Factor (GF) as it corresponds more to the natural development.

In *Mitoplinthuscaliginosus* (Fabricius, 1775) (subfamily Molytinae), after comparison of the growth factors 1.35, 1.4 and 1.5, the best approximation was found with a value of 1.4 for head capsule width (Sprick and Gosik 2014). This value agrees with Dyar’s ratio of 0.714. Rowe and Kok (1985) gave a Dyar’s ratio value for *Rhinocyllusconicus* (Frölich, 1792) (subfamily Lixinae) larvae of 0.65 (this agrees with a GF of 1.538). In agreement Leibee et al. (1980) determined the ratios of each instar of two populations of *Sitonahispidulus* (Fabricius, 1777) (subfamily Entiminae, tribe Sitonini) and reported Dyar’s values between 0.642 and 0.739. The median value of these data is by our calculation 0.6995 (GF = 1.43). These data show that there are rather different values for larval growth and that there are also differences between the growth of different larval instars.

For larval instar determination in *Tanymecus* we tested four values between 1.4 and 1.5 to achieve the best approximation of larval growth. We started with the L_1_ larva that we received from egg-laying of adult weevils (head width 0.38 mm) and calculated the subsequent instars with the selected GF values until 1.71 mm, the head width of the mature larvae, was achieved. For this procedure, five steps were needed. Higher GF values, as for example 1.538 in *Rhinocyllusconicus*, were excluded because of the reduced number of larval instars in this rather distantly related subfamily (Table 3).

From Table 2 it can easily be seen that both species have 5 larval instars. The best approximation is achieved with a GF of 1.44 in *Tanymecusdilaticollis* and 1.46 in *T.palliatus* (i.e. Dyar’s ratio of 0.694 and 0.685, respectively). The small difference may be due to the absent HW variation of the two available adult *T.dilaticollis* specimens that showed both the same value and hence do not represent the HW variation of the population. Furthermore it can be stated that the values of Catrinici (1944) are beginning to seem doubtful from the fourth larval instar onward.

For this approximation it is only necessary to know the head width of the L_1_ larva and that of the last instar. And the HW of the last instar can be assessed from the HW of the adult weevil, as it is shown in Table 2. In adults HW was always measured directly behind the eyes to avoid an excessive importance of prominent eyes, which could be a problem in genera such as*Strophosoma* Billberg, 1817 (see Gosik et al. 2017) or in species such as*Tanymecusdilaticollis*. Larval growth, number of larval instars and size of the adults’ head width (and therefore size of adults, too) are in a very close relationship to each other. The same may be true for the HW of the pupa.

An instar determination is also possible for *Graptustriguttatus*. According to Van Emden (1952), the head capsule width of the L_1_ larva is 0.34 mm (average of three larvae; Table 1). The application of a GF of 1.45 shows a good approximation with the measuring values given in Table 1: 0.34 mm × 1.45 (repeatedly) = 0.493 mm (L_2_), 0.715 mm (L_3_), 1.037 mm (L_4_) and finally 1.503 mm (L_5_). Thus, *Graptustriguttatus* has also 5 larval instars, and the premature larvae from Table 1 may represent L_2_ and L_4_ larvae. There is a great variation in adults’ head width in this species ranging from 1.05 mm to 1.5 mm (Table 1). This agrees with the span between the last larval instars and nearly achieves the supposed growth factor of 1.45, so that the assignment of a certain larva to the right instar is doubtful in extremely sized specimens, the more as head width variation becomes larger with instar and size. A similar size variation was observed in *Peritelussphaeroides* adults and *Philopedonplagiatum* larvae (Table 1).

In *Peritelussphaeroides* and *Philopedonplagiatum* an instar determination is impossible due to the absence of L_1_ head width data. It can only be concluded from the data for premature larvae in *Philopedonplagiatum* (Table 1) that these data represent the penultimate instar. Opposite to *Graptus* and *Tanymecus*, the HW of *Philopedon* adults is greater than in mature larvae. In *Peritelussphaeroides* the HW of adults is slightly smaller than in mature larvae, but not significantly.

**Table 1.** Head width measuring data of the species studied. Results in mm; ^n^ – number of specimens measured, in adults behind eyes; L_1_ – first instar larva; ML – mature larva; *: an assignment to this instar is doubtful. A transfer to ‘mature larvae’ would change the average value only slightly; **: data from Gosik and Sprick (2013). Data from literature in italics.

Species	Larval instars			Pupa	Adult
	L_1_ larvae	Premature larvae	Mature larvae		
***Graptustriguttatus*** (L_1_ data from Van Emden 1952)	*0.33*^1^; *0,34*^1^; *0.35*^1^	0.57^2^; 0.60^3^; 1.00^1^; 1.07^2^; 1.17^1^	1.37^3^; 1.40^2^; 1.43^1^; 1.47^2^; 1.50^6^; 1.53^4^; 1.55^1^	1.13^1^; 1.17^1^; 1.20^1^	1.05^1^; 1.15^1^; 1.2^1^; 1.25^3^; 1.3^3^; 1.4^1^; 1.45^1^; 1.5^1^
Mean value (x¯)	0.34	0.806	1.471	1.167	1.283
*** Peritelus sphaeroides ***	–	–	0.87^1^; 0.90^2^; 1.10^5^; 1.13^3^; 1.17^4^	1.00^1^; 1.05^1^	0.75^1^; 0.85^1^; 0.95^2^; 1.0^2^; 1.025^1^; 1.2^1^
Mean value (x¯)	–	–	1.083	1.025	1.025
*** Philopedon plagiatum ***	–	0.60^1^; 0.67^4^; 0.73^1^	0.97^1^;1.00^1^; 1.03^1^	–	1.05^1^; 1.1^2^; 1.225^1^; 1.25^1^; 1.375^1^; 1.4^2^
Mean value (x¯)	–	0.668	1.00	–	0.966
***Philopedonplagiatum*** (from Van Emden 1952)	–	0.57^2^; 0.61^1^; 0.63^1^; 0.64^1^; 0.68^1^; 0.72^1^; 0.75^1^*; 0.79^1^*	*0.86^1^; 0.89^2^; 0.96^2^; 1.00^1^; 1.07^2^; 1.08^1^; 1.1^2^; 1.14^1^; 1.17^1^; 1.18^1^; 1.21^2^*	–	–
Mean value (x¯)	–	0.662	1.056	–	–
*** Tanymecus dilaticollis ***	–	–	–	–	1.30^2^
Mean value (x¯)	–	–	–	–	1.30
*** Tanymecus palliatus ***	0.34^1^; 0.37^2^; 0.38^5^; 0.39^2^; 0.40^2^	–	1.62^1^; 1.70^1^; 1.80^1^	–	1.275^1^;1.425^1^; 1.5^1^; 1.55^1^; 1.6^1^
Mean value (x¯)	0.380	–	1.717	–	1.470

**Table 2.** Head width measuring data of *Tanymecus* larvae and adults for larval instar determination. *: collected together with *T.palliatus* pupae; **: calculated from the ratio of adult’s HW to HW of the mature larva.

Instar	Mean value (mm)	Specimens measured	Source
*** Tanymecus dilaticollis ***			
L_1_	0.35	12 larvae	Catrinici (1944)
L_2_	0.56	7 larvae	Catrinici (1944)
L_3_	0.77	5 larvae	Catrinici (1944)
L_4_	1.27	4 larvae	Catrinici (1944)
Mature larva	1.51**	–	calculated value
Adult weevil	1.30	2 adults	own data
*** Tanymecus palliatus ***			
L_1_	0.38	12 larvae	own data (see Tab. 1)
Mature larva*	1.71	3 larvae	own data (see Tab. 1)
Mature larva	1.71	5 larvae	Van Emden (1952)
Adult weevil	1.47	5 adults	own data

**Table 3.** Larval instar determination for *Tanymecusdilaticollis* and *T.palliatus*. All measuring data in mm; initial data bold, calculated data in italics; target data of the approximation bold and in italics.

*** Tanymecus palliatus ***			
Growth factor (to be tested)	1.40	1.45/1.46	1.50
L_1_ (measured)	**0.38**	**0.38**	**0.38**
L_2_ (calculated)	*0.532*	*0.551/0.555*	*0.57*
L_3_ (calculated)	*0.745*	*0.799/0.810*	*0.855*
L_4_ (calculated)	*1.042*	*1.158/1.183*	*1.283*
L_5_ (calculated)	*1.460*	*1.680/1.727*	*1.924*
Mature larva (measured)	**1.71**	**1.71**	**1.71**
Tanymecusdilaticollis*			
Growth factor (to be tested)	1.40	1.44/1.45/1.46	1.50
L_1_ (measured)	**0.35**	**0.35**	**0.35**
L_2_ (measured)	0.56	0.56	0.56
L_2_ (calculated)	*0.49*	*0.504/0.508/0.511*	*0.525*
L_3_ (measured)	0.77	0.77	0.77
L_3_ (calculated)	*0.686*	*0.726/0.736/0.746*	*0.7875*
L_4_ (measured)	1.27	1.27	1.27
L_4_ (calculated)	*0.960*	*1.045/1.067/1.089*	*1.181*
L_5_ (calculated)	*1.345*	*1.505/1.55/1.590*	*1.772*
Mature larva (calculated; see Tab. 1)	***1.51***	***1.51***	***1.51***

*: measuring data from Catrinici (1944).

**Table 4.** Number of setae in mature larvae of *Graptustriguttatustriguttatus* (a), *Peritelussphaeroides* (b), *Philopedonplagiatum* (c), *Tanymecuspalliatus* (d).

Part of body	Setae	a	b	c	d
Prothorax	*prns*	8	9	9	11
	*ps*	2	2	2	2
	*eus*	1	1	1	1
	*pda*	6	4	9	6
Meso-, metathorax	*prs*	1	1	1	1
	*pds*	4	4	4	4
	*as*	1	1	2	2
	*ss*	3	2	3	2
	*eps*	1	1	1	1
	*ps*	1	1	1	1
	*eus*	1	1	1	1
	*pda*	6	4	9	6
Abdominal segments 1–8	*prs* 1-7	1	1	1	1
	*prs* 8	1	1	1	1
	*pds* 1-7	5	5	5	5*
	*pds* 8	4	4	5	4
	*ss* 1-7	2	2	2	1
	*ss* 8	1	1	1	0
	*eps* 1-7	2	2	3-4	2
	*eps* 8	2	2	2	2
	*ps* 1-7	2	2	2	2
	*ps* 8	2	2	2	2
	*lsts* 1-7	1	1	1	1
	*lsts* 8	1	1	1	1
	*eus* 1-7	2	2	2	2
	*eus* 8	2	2	2	2
Abdominal segment 9	*ds*	3	4	2	3
	*ps*	2	1	1	1
	*sts*	2	2	2	2
Abdominal segment 10	*ts*	2	2	0	3
Head	*des*	4	4	5	4
	*fs*	2	2	3	2
	*ls*	2	2	2	2
	*pes*	3	7	4	5
	*ves*	1	0	1	2
Mouthpart	*cls*	2	2	2	2
	*lrs*	3	3	3	3
	*mes*	2	2	2	2
	*als*	3	3	3	4
	*ams*	3	3	3	3
	*mds*	2	1	2	2
	*des*	7	7	8	7
	*ves*	4	4	4	4
	*pfs*	2	2	2	2
	*stps*	1	1	1	1
	*pbs*	1	1	1	1
	*mps*	1	1	1	1
	*pms*	3	3	3	3
	*prms*	1	1	1	1
	*ligs*	3	2	2	2

* only abdominal segment 7 with 6 *pds*.

## Discussion

### I. Significance of morphological features of larvae for the relationship between genera and higher taxa

*Philopedon* belongs to genera with abdominal type ‘B’ larvae together with *Strophosoma* and *Tanymecus* (Van Emden 1950, 1952). The main feature is the presence of a flattened dorso-ventral abdominal segment 9. Furthermore this feature (unique among weevils) may suggest an unknown kind of relationship between these genera (Gosik et al. 2017). Nevertheless, there are some morphological differences between larvae of type B, for example, the abdominal segment 10 is almost covered by segment 9 in *Philopedon*, whereas segment 10 is integrated with segment 9 and forms a sclerotized ventral surface in *Strophosoma* and *Tanymecus*. The dorsal lobe of segment 9 is largest in *Strophosoma*, whereas the ventral lobe is largest in *Tanymecus*.

According to Van Emden (1952), the chaetotaxy of the mature larva of *Tanymecuspalliatus* is only slightly different from L_1_, namely: only six setae instead seven on the pedal lobe and lack of microseta *des_4_*. It is worth mentioning that the proportion between (and arrangement of) setae shows the same order on L_1_as on mature larvae, a finding that is not common in Entiminae (Fidler 1936; Gosik and Sprick 2012b; Gosik et al. 2016, 2017). To the characters listed by Van Emden (1952)as typical for the genus *Tanymecus* we can add that the clypeus is twice as long as labrum (Fig. 66).

The mature larva of *Philopedonplagiatum* is described by Van Emden (1952). The only exception is *cls*: rather short on L_1_ versus extremely long on the mature larva; *cls*as long as*lrs_1_* is not observed in other Entiminae.

In *Graptustriguttatus* the shape of the body, number and proportion of setae on L_1_ (according to Van Emden 1952) and of the mature larva (present paper) are almost identical. The tribe Byrsopagini (with the genus *Graptus* Schönherr, 1823) is currently removed from the subfamily Molytinae Schönherr, 1823 to the subfamily Entiminae Schönherr, 1823 (Thompson 1992; Marvaldi 1997, 1998a; Alonso-Zarazaga and Lyal 1999; Löbl and Smetana 2013). From two apomorphies, most typical for Entiminae (4 *vms* and cushion-like sensorium) in the sense of Marvaldi (1997, 1998a, 1998b, 2003), the larva of *Graptus* possesses only this first character (4 *vms*). But 4 *vms*, observed constantly on larvae of all Entiminae, were also recorded on several other weevil taxa (e.g., Tychinii Gistel, 1848; Skuhrovec et al. 2015). On the other hand, the number of setae and shape of the larval body of *G.triguttatus* seem to be more similar to the Entiminae than to the Molytinae. But the fact that different larval stages and the pupae were found between the roots of dicotyledonous plants, outside of any plant tissues, supports the placement of *Graptus* (and Byrsopagini) in the subfamily Entiminae (Thompson 1992; Marvaldi 1997, 1998a; Alonso-Zarazaga and Lyal 1999); even taking into account some small differences between the description of the mature larva of *G.triguttatus* presented by us and descriptions of L_1_ presented by Van Emden (1952) and Marvaldi (1998a) (e.g., the lack of *ts* on L_1_ versus lateral lobes of mature larva with a pair of *ts*). On the other hand, the rest of characters listed by Marvaldi (1998a) (e.g., 1 *as*; 4–6 *pds* on Abd. 8; sensillum cluster between *mes_1_* and *mes_2_*; mandibles with bidentate apex; four anal lobes) have been mentioned in both descriptions.

From morphological data, the taxonomic position of Byrsopagini is apparently not so clear, but its placement within Entiminae is more plausible than in Molytinae; this is in line with the results of the cladistic and phylogenetic analyses performed by Marvaldi (1997, 1998a) and Stüben et al. (2015).

**Figures 71–76.**
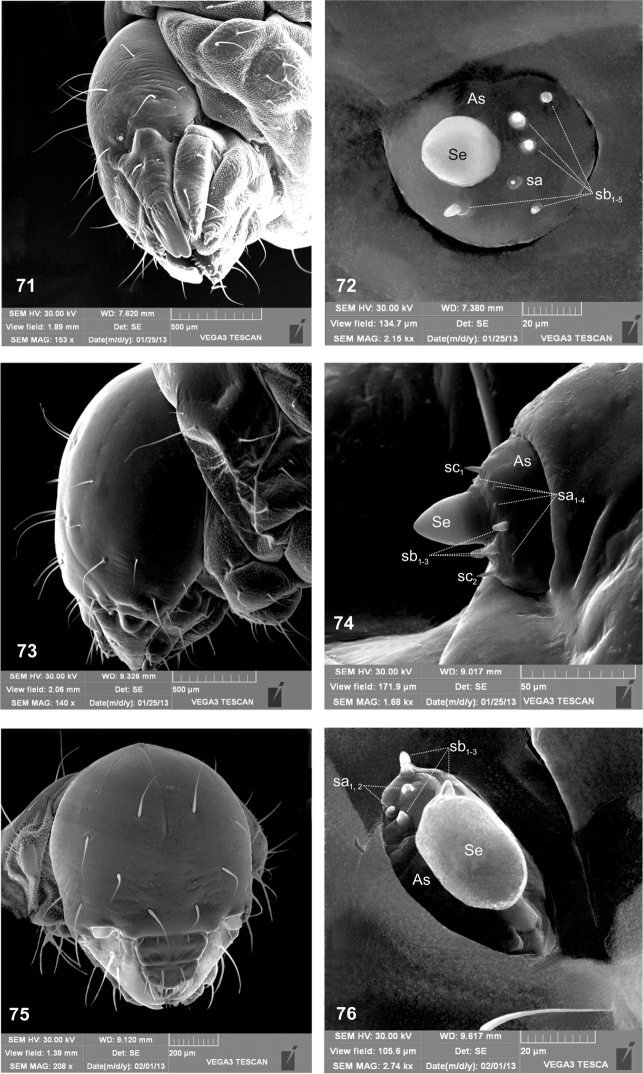
*Graptustriguttatustriguttatus*, mature larva. **71** Head **72** Antenna *Mitoplinthuscaliginosus* mature larva **73** Head **74** Antenna, *Philopedonplagiatum* mature larva **75** Head **76** Antenna. Abbreviations: as – antennal segment, Se – sensorium, sa – sensillum ampullaceum, sb – sensillum basiconicum, sc – sensillum chaeticum.

Moreover, a detailed analysis of the structure of antennae disclosed only ostensible similarities between *Graptus* and Molytinae, e.g. *Mitoplinthuscaliginosus* described by Sprick and Gosik (2014). First of all, the larva of *M.caliginosus* shows a larger number and variety of sensilla than the *Graptus* larva. The shape of the antennal sensorium is quite variable in both genera as well. Finally, the sensorium on *Mitoplinthus* is elongate and pointed, whereas it is stout and rounded in *Graptus* (Figs 71–76). The presence of a conical instead of a cushion-like sensorium in *Graptus* larvae is difficult to explain. Van Emden (1952) described the larva of *Philopedonplagiatum* with “broader and more rounded” sensoria. The same type of structure was found in larvae obtained during this study (Fig. 51).

**Figures 77, 78.**
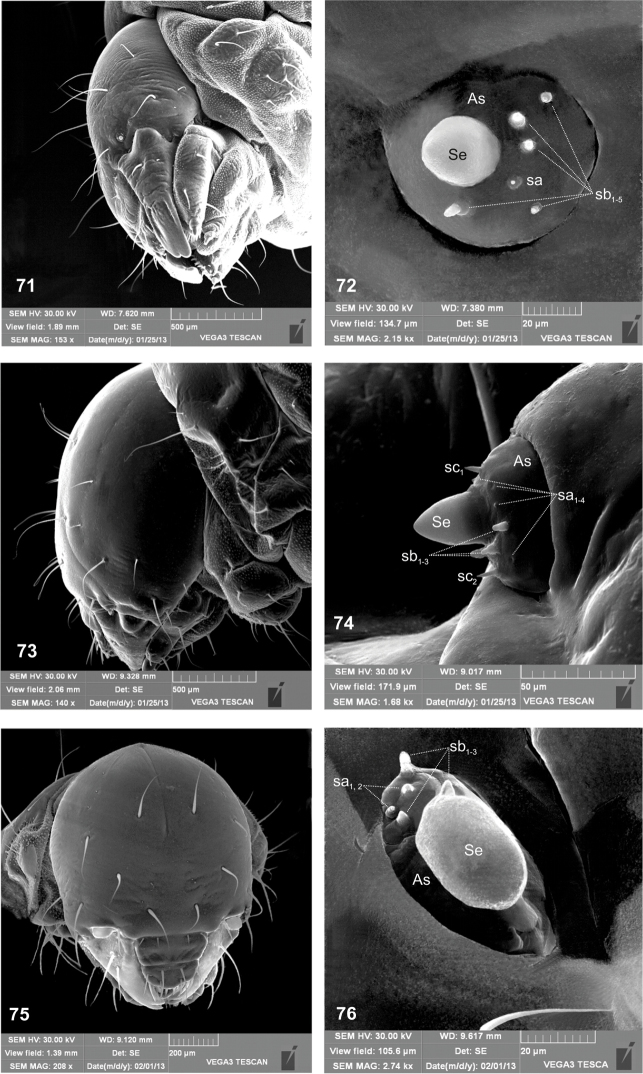
*Graptustriguttatustriguttatus*, mature larva. **77** Thoracic spiracle **78** Abdominal spiracle.

Van Emden (1950) did not recognize any kind of spiracles of the *Graptustriguttatus* larva, and finally marked them as “?”. At first view they could be recognized as bicameral, but under high magnification (40×) they turned to appear annularly (Figs 77, 78).

The description of the pupa of *Graptustriguttatus* did not provide any relevant features that could confirm a replacement of this genus in the Entiminae. It seems to be especially difficult to distinguish them from other subfamilies due to the large diversity of both, shape and chaetotaxy, and the absence of features strictly characteristic of Entiminae pupae (Gosik and Sprick 2012a, 2013). The absence of urogomphi in pupae of *G.triguttatus* corresponds with pupae of some species of *Otiorhynchus* Germar, 1822 with strongly reduced urogomphi (Gosik and Sprick 2012a) and with the pupa of *Liophloeustessulatus* (Müller, 1776), which does not bear urogomphi at all. Unfortunately, pupae of Molytinae are also characterized by variable development of urogomphi: they are well developed in the genera *Pissodes* Germar, 1817 and *Trachodes* Germar, 1824 (Scherf 1964) but almost absent in *Homalinotus* Schönherr, 1823 (De Oliveira Lira et al. 2017).

### II. Some aspects of biology

*Graptustriguttatus*. In January and April 2013 many larvae of different instars were found, and most of them were already mature (Figs 85–87), which indicates hibernation in a higher instar and no development in this early time of the year. In May and June the proportion of mature larvae continued to grow, and pupae were also recorded. We conclude that teneral adults will be present from July onward. The biology is nevertheless poorly known. Dudich (1921) stated that adult weevils are mainly present from mid-April to mid-May. These findings are difficult to explain. If the species directly overwinters in soil after emerging from the pupa, the presence of winter larvae of mainly higher instars is unexpected, and if the species appears on the soil surface in summer after reproduction, then the maximum of adults in April and May is hard to explain. It seems probable that there is a high degree of overlap between different generations; the life-cycle has still to be clarified.

*Peritelussphaeroides*. According to Hoffmann (1963) and Dieckmann (1980), eggs are deposited in April and May and the new generation of adults emerges from June to August. Our data, with mature larvae from the end of August (Figs 83, 84), November and December (most larvae) until March of the following year, and two pupae found in December (Gosik and Sprick 2013), may suggest that there is a much longer period of larval development than given by the sources cited above. Probably there is a degree of overlap between the generations, as it was suggested for *Graptustriguttatus* and as it was often found in *Otiorhynchus* species (e.g., Gosik et al. 2016). Apparently, there is a need for regular search of larvae and pupae in the field to clarify the life-cycle.

**Figures 79–87.**
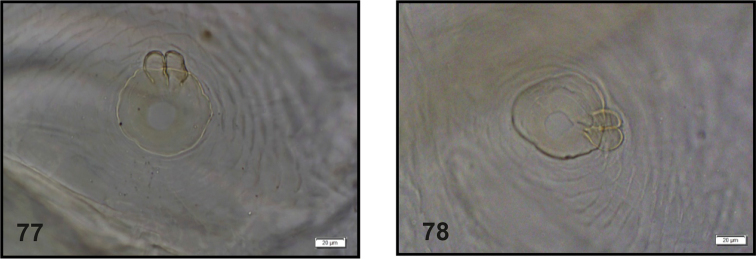
Habitats, host plants, digging sites and larvae of soil-dwelling weevils. **79** Habitat of *Philopedonplagiatum* in Southwestern Denmark, sandy sea shore of the North Sea **80** Habitat of *P.plagiatum* in Hannover-Vahrenheide, Kugelfangtrift, sand path with *Plantagolanceolata***81** Digging site of *P.plagiatum*, sandy soil with *P.lanceolata*at the Kugelfangtrift **82** Larva of the penultimate instar of *P.plagiatum*, found in the field in October, just molted, with light head capsule **83, 84** Larva of *Peritelussphaeroides*, bred in flowerpots with *Euonymusfortunei* in a climate chamber **85, 86** Host plant (*Plantagolanceolata*) and digging site of *Graptustriguttatustriguttatus* under *P.lanceolata* in an occasionally mown meadow of the JKI area in Braunschweig **87** Mature or penultimate instar larva of *Graptustriguttatustriguttatus*, found in the field in Braunschweig in April.

*Philopedonplagiatum*. A description of the pupa is not available, even though the species attracted attention occasionally in beet fields, horticultural crops and pine plantations in sandy areas (Figs 79–82) (e.g., Dieckmann 1980; Brendler et al. 2008). Although teneral adults were found mainly in April (Dieckmann 1980), the period of pupation cannot be ascertained from this fact. In several species, such as*Otiorhynchusraucus* or *O.singularis*, pupation occurs in mid-summer and adults overwinter with smooth cuticula in their pupal chambers until next spring (Gosik et al. 2017). Morris (1987) found a young adult weevil with mandibular appendages in September. The fact of a long development and overlap of generations, as also suggested for the two species treated before, *Graptustriguttatus* and *Peritelussphaeroides*, can be directly concluded from the data on *P.plagiatum* given by Dieckmann (1980). Morris’ observations support these findings; he also stated that the species does not have a simple life-cycle, and he already supposed that it develops over two years. This seems to be probable and the best explanation for the data presented previously.

## Supplementary Material

XML Treatment for Graptus triguttatus triguttatus

XML Treatment for Peritelus sphaeroides

XML Treatment for Philopedon plagiatum

XML Treatment for Tanymecus palliatus

## References

[B1] Alonso-Zarazaga MA Lyal CHC (1999) A World Catalogue of Families and Genera of Curculionoidea (Insecta: Coleoptera) (Excepting Scolytidae and Platypodidae). Entomopraxis, Barcelona, Spain, 315 pp.

[B2] Alonso-Zarazaga MA Barrios H Borovec R Bouchard P Caldara R Colonnelli E Gültekin L Hlaváč P Korotyaev B Lyal CHC Machado A Meregalli M Pierotti H Ren L Sánchez-Ruiz M Sforzi A Silfverberg H Skuhrovec J Trýzna M Velázquez de Castro AJ Yunakov NN (2017) Cooperative Catalogue of Palaearctic ColeopteraCurculionoidea Sociedad Entomológica Aragonesa, Monografias electrónicas SEA, 8. http://www.sea-entomologia.org [accessed November 10, 2017]

[B3] Anderson WH (1947) A terminology for the anatomical characters useful in the taxonomy of weevil larvae. Proceedings of the Entomological Society of Washington 49(5): 123–132.

[B4] Brendler F Holtschulte B Rieckmann W (2008) Grauer Kugelrüssler *Philopedonplagiatus* Schaller (Syn. *Cneorhinus*). Zuckerrübe, Krankheiten, Schädlinge, Unkräuter. Agroconcept, Bonn, Gelsenkirchen, 130.

[B5] Catrinici C (1944) Die Bestimmung der Larvenstadien des Rüsselkafers Tanymecusdilaticollis Gyll. Bulletin Section Scientia Academia Romania 26: 626–635.

[B6] Dieckmann L (1980) Beiträge zur Insektenfauna der DDR: Coleoptera – Curculionidae (Brachycerinae, Otiorhynchinae, Brachyderinae). Beiträge zur Entomologie 30(1): 145–310.

[B7] Dieckmann L (1983) Beiträge zur Insektenfauna der DDR: Coleoptera – Curculionidae (Tanymecinae, Leptopiinae, Cleoninae, Tanyrhynchinae, Cossoninae, Raymondionyminae, Bagoinae, Tanysphyrinae). Beiträge zur Entomologie 33(2): 257–381.

[B8] Dudich E (1921) Zur Biologie des *Alophustriguttatus* F. Entomologische Blätter 17(4–6): 62–64.

[B9] Dyar HG (1890) The number of molts of lepidopterous larvae. Psyche 5: 420–422. 10.1155/1890/23871

[B10] Emden FI Van (1950) Eggs, egg-laying habits and larvae of short-nosed weevils. Eighth International Congress of Entomology, Stockholm 1948: 365–372.

[B11] Emden FI Van (1952) On the taxonomy of *Rhynchophora* larvae: Adelognatha and Alophinae (Insecta: Coleoptera) Proceedings of the Zoological Society of London 122(3): 651–795. 10.1111/j.1096-3642.1952.tb00248.x

[B12] Fidler H (1936) On the first instar larvae of some species of *Otiorrhynchus* found on strawberries, with notes on their biology. Bulletin of Entomological Research 27: 369–376. 10.1017/S0007485300058211

[B13] Gosik R Sprick P (2012a) Morphology and identification of the pupae of seven species of the genus Otiorhynchus Germar, 1822 (Coleoptera, Curculionidae, Otiorhynchini). Zootaxa 3483: 39–57. 10.11646/zootaxa.3731.4.2

[B14] Gosik R Sprick P (2012b) Larval morphology of *Otiorhynchusligustici*, *O.porcatus* and *O.salicicola* (Coleoptera, Curculionidae, Otiorhynchini). Deutsche Entomologische Zeitschrift 59: 301–316. 10.1002/mmnd.201200025

[B15] Gosik R Sprick P (2013) Morphology and identification of the pupae of several species of soil-dwelling broad-nosed weevils from Central Europe (Coleoptera, Curculionidae, Entiminae). Zootaxa 3731(4): 445–472. Incl. Erratum: Zootaxa 3745(2): 299–300. 10.11646/zootaxa.3731.4.2 25277585

[B16] Gosik R Sprick P Skuhrovec J Deruś M Hommes M (2016) Morphology and identification of the mature larvae of several species of the genus *Otiorhynchus* (Coleoptera, Curculionidae, Entiminae) from Central Europe with an update of the life history traits. Zootaxa 4108(1): 1–67. 10.11646/zootaxa.4108.1.1 27394846

[B17] Gosik R Sprick P Czerewko K (2017) Morphology of the larvae of three Central European *Strophosoma* Billberg, 1820 (Coleoptera, Curculionidae, Entiminae) species. Deutsche Entomologische Zeitschrift 64: 27–42. 10.3897/dez.64.11446

[B18] Hoffmann A (1963) Entomologie appliquée à l’agriculture. Tome I. Coléoptères. Second volume. Phytophagoidea (suite et fin) (Chrysomelidae – Curculionidae – Attelabidae – Scolytidae et Platypodidae). Sous-famille des Otiorrhynchinae. Paris, Masson, 878–1360.

[B19] Leibee GL Pass BC Yeargan KV (1980) Instar determination of Clover Root Curculio, Sitonahispidulus (Coleoptera: Curculionidae). Journal of the Kansas Entomological Society 53(3): 473–475.

[B20] Löbl I Smetana A (2013) Catalogue of Palaearctic Coleoptera. Vol. 8. Curculionoidea II. Brill, Leiden/Boston, 700 pp.

[B21] Marvaldi AE (1997) Higher level phylogeny of Curculionidae (Coleoptera: Curculionoidea) based mainly on larval characters, with special reference to broad-nosed weevils. Cladistics 13: 285–312. 10.1111/j.1096-0031.1997.tb00321.x 34911227

[B22] Marvaldi AE (1998a) Larvae of Entiminae (Coleoptera: Curculionidae): tribal diagnoses and phylogenetic key, with a proposal about natural groups within Entimini. Entomologica Scandinavica 29: 89–98. 10.1163/187631298X00212

[B23] Marvaldi AE (1998b) Larvae of South American Rhytirrhininae (Coleoptera: Curculionidae). The Coleopterists Bulletin 52(1): 71–89.

[B24] Marvaldi AE (1999) Morfología larval en Curculionidae. Acta Zoológica Lilloana 45: 7–24.

[B25] Marvaldi AE (2003) Key to larvae of the South American subfamilies of weevils (Coleoptera, Curculionoidea). Revista Chilena de Historia Natural 76: 603–612. 10.4067/S0716-078X2003000400005

[B26] May BM (1994) An introduction to the immature stages of Australian Curculionoidea. In: Zimmerman EC (Ed.) Australian Weevils.Volume II. Brentidae, Eurhynchidae, Apionidae and a chapter on immature stages by Brenda May. CSIRO, Melbourne, 1–755.

[B27] Morris MG (1987) The distribution and ecology of *Philopedonplagiatus* (Schaller) (Col.: Curculionidae), with particular reference to inland records. Entomologist’s Record and Journal of Variation 99: 11–20.

[B28] Oliveira Lira A De Rosado-Neto GH Marques MI Oliveira De Sous W (2017) A first description of the larva and pupa of the coconut palm borer *Homalinotusdepressus* (Linnaeus, 1758) (Coleoptera: Curculionidae: Molytinae) and a discussion about the terminology for immature forms. Zootaxa 4311(4): 589–599. 10.11646/zootaxa.4311.4.10

[B29] Rosenstiel RG (1987) Larval taxonomy of some Polydrosinae and Entiminae (Coleoptera: Curculionidae). Miscellaneous Publications of the Entomological Society of America 67: 1–64.

[B30] Rowe DJ Kok LT (1985) Determination of larval instars, and comparison of field and artificial diet-reared larval stages of *Rhinocyllusconicus* (Col: Curculionidae). Virginia Journal of Science 36(4): 277–280.

[B31] Scherf H (1964) Die Entwicklungsstadien der mitteleuropäischen Curculioniden (Morphologie, Bionomie, Ökologie). Abhandlungen der Senckenbergischen Naturforschenden Gesellschaft 506: 1–335.

[B32] Skuhrovec J Gosik R Caldara R Košťál M (2015) Immatures of Palaearctic species of the weevil genus *Sibinia* (Coleoptera, Curculionidae): new descriptions and new bionomic data with suggestions on their potential value in a phylogenetic reconstruction of the genus. Zootaxa 3955(2): 151–187. 10.11646/zootaxa.3955.2.1 25947846

[B33] Sprick P Gosik R (2014) Biology and morphology of the mature larva of *Mitoplinthuscaliginosuscaliginosus* (Curculionidae, Molytinae). Snudebiller. Studies on Taxonomy, Biology and Ccology of Curculionoidea 15(229): 1–10. https://www.curci.de/?beitrag=229

[B34] Stüben PE Schütte A Bayer C Astrin JJ (2015) The Molecular Weevil Identification Project (Coleoptera: Curculionoidea), Part II. Towards an Integrative Taxonomy. Snudebiller. Studies on taxonomy, biology and ecology of Curculionoidea 16(237): 1–294. [including No. 238: table with phylogenetic relations. Curculio–Institute, Mönchengladbach. https://www.curci.de/?beitrag=237 and https://curci.de/data/snudebiller/sn16/btr_238/16g_text238.pdf]

[B35] Thompson RT (1992) Observations on the morphology and classification of weevils (Coleoptera, Curculionoidea) with a key to major groups. Journal of Natural History 26: 835–891. 10.1080/00222939200770511

[B36] Trnka F Stejskal R Skuhrovec J (2015) Biology and morphology of immature stages of *Adosomusroridus* (Coleoptera: Curculionidae: Lixinae). Zootaxa 4021: 433–446. 10.11646/zootaxa.4021.3.3 26624140

[B37] Zacharuk RY (1985) Antennae and sensilla. In: Kerkut GA, Gilbert LI (Eds) Comparative Insects Physiology, Chemistry and Pharmacology 6. Pergamon Press, Oxford, 69 pp.

[B38] Znamenskij AV (1927) Posobie dlja Proisvodstva Obsledovanija Entomofauni Potschvi 595 (7). S.S.U. Pravlenija Sacharotresta, Kiew, 3–72. [extract pp. 22–24] [In Russian]

